# Heterogeneous associations between smoking and a wide range of initial presentations of cardiovascular disease in 1 937 360 people in England: lifetime risks and implications for risk prediction

**DOI:** 10.1093/ije/dyu218

**Published:** 2014-11-20

**Authors:** Mar Pujades-Rodriguez, Julie George, Anoop Dinesh Shah, Eleni Rapsomaniki, Spiros Denaxas, Robert West, Liam Smeeth, Adam Timmis, Harry Hemingway

**Affiliations:** ^1^Farr Institute of Health Informatics Research, University College London, London, UK, ^2^Department of Non-Communicable Disease Epidemiology, London School of Hygiene and Tropical Medicine, London, UK and ^3^National Institute for Health Research, Biomedical Research Unit, Barts Health NHS Trust, London, UK

**Keywords:** Association study, cardiovascular outcomes, epidemiology, initial presentation, lifetime risks, primary prevention, smoking, risk prediction, risk stratification

## Abstract

**Background** It is not known how smoking affects the initial presentation of a wide range of chronic and acute cardiovascular diseases (CVDs), nor the extent to which associations are heterogeneous. We estimated the lifetime cumulative incidence of 12 CVD presentations, and examined associations with smoking and smoking cessation.

**Methods** Cohort study of 1.93 million people aged ≥30years, with no history of CVD, in 1997–2010. Individuals were drawn from linked electronic health records in England, covering primary care, hospitalizations, myocardial infarction (MI) registry and cause-specific mortality (the CALIBER programme).

**Results** During 11.6 million person-years of follow-up, 114 859 people had an initial non-fatal or fatal CVD presentation. By age 90 years, current vs never smokers’ lifetime risks varied from 0.4% vs 0.2% for subarachnoid haemorrhage (SAH), to 8.9% vs 2.6% for peripheral arterial disease (PAD). Current smoking showed no association with cardiac arrest or sudden cardiac death [hazard ratio (HR) = 1.04, 95% confidence interval (CI) 0.91–1.19).The strength of association differed markedly according to disease type: stable angina (HR = 1.08, 95% CI 1.01–1.15),transient ischaemic attack (HR = 1.41, 95% CI 1.28-1.55), unstable angina (HR = 1.54, 95% CI 1.38–1.72), intracerebral haemorrhage (HR = 1.61, 95% CI 1.37–1.89), heart failure (HR = 1.62, 95% CI 1.47–1.79), ischaemic stroke (HR = 1.90, 95% CI 1.72–2.10), MI (HR = 2.32, 95% CI 2.20–2.45), SAH (HR = 2.70, 95% CI 2.27–3.21), PAD (HR = 5.16, 95% CI 4.80–5.54) and abdominal aortic aneurysm (AAA) (HR = 5.18, 95% CI 4.61–5.82). Population-attributable fractions were lower for women than men for unheralded coronary death, ischaemic stroke, PAD and AAA. Ten years after quitting smoking, the risks of PAD, AAA (in men) and unheralded coronary death remained increased (HR = 1.36, 1.47 and 2.74, respectively).

**Conclusions** The heterogeneous associations of smoking with different CVD presentations suggests different underlying mechanisms and have important implications for research, clinical screening and risk prediction.

**Key Messages**

- This paper presents a population-based cohort analysis of contemporary electronic health records from more than 1.9 million adults with more than 100 000 initial presentations of 12 different non-fatal and fatal, acute and chronic cardiovascular diseases.
- We demonstrate that current smoking has highly heterogeneous associations with different types of cardiovascular disease.
- In particular we report: associations with chronic conditions which have seldom been studied in large-scale cohorts, such as heart failure (moderate association), peripheral arterial disease (very strong association) and chronic stable angina (weak association); and lack of association with sudden cardiac death and ventricular arrhythmia.
- Our findings suggest differences in underlying disease mechanisms, and have important implications for risk prediction, clinical practice and aetiological research.

## Introduction

Cigarette smoking is known to be a major modifiable risk factor for cardiovascular diseases (CVDs). Its relationship with cardiovascular diseases and the reduction in risk following smoking cessation^1^ and implementation of comprehensive smoke-free legislations is well documented.^2–4^ The focus of previous smoking research has generally been on final manifestations (CVD mortality)^5^ or on one or two non-fatal diseases, usually acute myocardial infarction (MI) and stroke.^4^^,^^6^ Other CVDs, for example heart failure^7^^,^^8^ or peripheral arterial disease (PAD),^9^ have been less commonly studied and the initial lifetime presentation of a wide range of acute and chronic, non-fatal and fatal CVDs has not been investigated in the same study population. In the 21st century, with rapid declines in the incidence of MI and stroke,^10^^,^^11^ chronic conditions such as PAD, heart failure and stable angina are becoming common initial presentations of CVD. Studying and comparing the initial presentation of a wide range of CVDs in the same population has been difficult because of the need for large cohorts with detailed clinical follow-up, covering hospital and ambulatory care. Recently, it has been suggested that linked electronic health record (EHR) data might provide the statistical scale and clinical resolution necessary for this research.^12^

Fundamental, inter-related questions concerning disease mechanism, public health and risk prediction remain unanswered and are addressed as the aims of the present study. First, what is the lifetime risk of current and ex- smoking for each disease? Lifetime risk estimates have been recently reported for aggregates of risk factors (not specific to smoking status) and aggregates of coronary heart disease (CHD) and CVD,^13^ but not for a wide range of specific cardiovascular phenotypes. Second, to what extent do smoking associations differ according to each specific CVD? Some variation in associations between smoking and different cardiovascular phenotypes is expected, given that smoking induces acute responses, including increases in blood pressure, heart rate or pro-thrombotic state; and chronic adaptation through increases in levels of low-density lipoprotein cholesterol, fibrinogen and platelet aggregation.^14–16^ For cancers, aetiological insights have come from the observation that smoking has no association with some (e.g. glioma), modest with others (e.g. stomach or kidney cancer relative risks for current vs non-smokers are 1.5–2.0) and very strong for lung cancer (relative risks of 15–30).^17^ As in cancer research, the study of heterogeneity in associations across CVD phenotypes could provide important aetiological insights and guide the design and interpretation of other studies. Third, how does the smoking effect for each CVD differ among men and women,^6^ at older ages, or among people with hypertension or diabetes? Fourth, what is the population-attributable fraction (PAF) for each disease and what is the contemporary relevance of clinically recorded smoking data in the light of recent policy such as financial rewards in primary care for smoking assessment and public smoking bans? Fifth, if smoking does have disease-specific associations, what are the implications for risk prediction for primary prevention? Currently used tools are based on a common estimate of smoking association with aggregates of CVD or CHD. However, because initial occurrence of one CVD strongly influences the development of another [e.g. transient ischaemic attack (TIA) predisposes to MI and stroke), the ability to predict different forms of CVD more precisely might offer the potential for earlier initiation of preventive therapies.

We addressed these questions using a contemporary cohort^18–20^ based on linked EHRs across primary care, secondary care, disease registry and death records of patients in England, with 6 years of median follow-up.

## Methods

### Study population

A cohort of 1 937 360 patients drawn from individuals registered in the general practices in England contributing with data to the CALIBER programme, between January 1997 and March 2010, was analysed. Patient data were linked across four data sources (Appendix 1.1, see Supplementary data available at *IJE* online); the Clinical Practice Research Datalink (CPRD); the Myocardial Ischaemia National Audit Project registry (MINAP); Hospital Episodes Statistics (HES); and the Office of National Statistics (ONS).^18^ Patients were eligible for inclusion when they were aged ≥30 years and had been registered in a practice meeting research data recording standards for at least 1 year. Patients with missing record of sex, those with history of CVD and those pregnant within 6 months of the eligibility date were excluded (Appendix 1.2, see Supplementary data available at *IJE* online).

### Smoking status

Patient self-reported smoking status was prospectively collected and coded by general practitioners or practice nurses on the date of consultation in CPRD. The most recent smoking record before study entry was used to classify individuals as never, ex- or current smokers, and those identified as current smokers with no smoking record within the 3 years before study entry were reclassified as having missing smoking data. Never smokers who had a record of smoking at any time before baseline were reclassified as ex-smokers. Ex-smokers were grouped into categories of time since quitting (<2, 2–5, 5–10, >10 years and missing) using the date of smoking interruption recorded in CPRD on or before study entry, which was available for 33.7% of ex-smokers. The median time between recorded baseline status and study entry was 1.0 year.

### Covariates

Covariates considered in the analysis were: sex, age, index of multiple deprivation, diabetes mellitus, body mass index (BMI), systolic blood pressure (SBP), total and high-density lipoprotein (HDL) cholesterol and medication use (blood pressure-lowering drugs, statins, oestrogen oral contraceptives and hormone replacement therapy). Baseline covariates were defined as the most recent measurement (or prescription) recorded in CPRD up to 1 year before study entry. Patients were defined as diabetic if they had a diagnosis or prescription for diabetes prior to the index date. Covariate definitions can be found at [http://www.caliberresearch.org/portal/].

### Endpoints

The primary endpoints were the initial presentation of non-fatal or fatal CVD across data sources. Diseases studied were: stable angina, unstable angina, MI, unheralded coronary death, heart failure; a composite of ventricular arrhythmia, cardioversion, cardiac arrest or sudden cardiac death (CA-SCD); TIA, ischaemic stroke, subarachnoid haemorrhage (SAH), intracerebral haemorrhage, PAD and abdominal aortic aneurysm (AAA). Secondary endpoints were: composite CVD (all cardiovascular endpoints except stable angina); ST-elevation MI (STEMI); and non-ST-elevation MI (NSTEMI). Diagnosis codes for each endpoint can be found at [http://www.caliberresearch.org/portal/] (Appendix 1.3, see Supplementary data available at *IJE* online).

### Statistical analysis

Patient follow-up was started on the date on which all eligibility criteria were met after 1 January 1997 and was censored on the date of first CVD presentation, death from other causes, last data collection from CPRD (25 March 2010) or deregistration from the practice, whichever occurred first. For each CVD, the lifetime cumulative incidence was estimated with Cox proportional hazard models adjusted for the competing risk of initial presentation with another CVD or death from other causes, using age as the time scale. In primary analyses, the relationship between current vs never smokers for each endpoint was assessed using age-adjusted, multiply imputed Cox proportional hazard models. The baseline hazard function of each model was stratified by sex and practice. Never smokers were the reference category. Multiple imputation was used to replace missing values in exposure and in risk factors. It was implemented using the *mice*^21^ algorithm in the statistical package R. Imputation models were estimated separately for men and women and included: (i) all the baseline covariates used in the main analysis (age, quadratic age, diabetes, smoking, systolic blood pressure, total cholesterol, HDL cholesterol, index of multiple deprivation); (ii) prior (between 1 and 4 years before study entry) and post (between 0 and 1 year after study entry) averages of continuous covariates in the main analysis; (iii) baseline measurements of covariates not considered in the main analysis (diastolic blood pressure, alcohol intake, white cell count, haemoglobin, creatinine, alanine transferase); (iv) baseline medications (statins, blood pressure-lowering medication, low-dose aspirin, loop diuretics, oestrogen oral contraceptives and hormone replacement therapy); (v) coexisting medical conditions (history of depression, cancer, renal disease, liver disease and chronic obstructive pulmonary disease); and (vi) the Nelson-Aalen hazard and the event status for each of the 12 endpoints analysed.^22^ Non-normally distributed variables were log-transformed for imputation and exponentiated back to their original scale for analysis. Five multiply imputed datasets were generated, and Cox models were fitted to each dataset. Coefficients were combined using Rubin’s rules. The Kolmogorov–Smirnov test was used to compare the distribution of observed vs imputed log-transformed covariates.

Assuming independence between initial presentations, heterogeneity in associations across CVD endpoints was assessed using the τ^2^ statistic.^23^ PAFs associated with current and former smoking were calculated using the Stata command *punafcc*. The clinical utility of smoking to predict the risk of CVD was assessed by estimating the improvement in risk discrimination (difference in the C-index) resulting from including smoking status into a Cox proportional hazard model with only age and sex, among patients aged 40–74 years (Vascular Health Screening target group in England).

In secondary analyses, we evaluated effect modification by sex and baseline age, diabetes, hypertension, implementation of the English smoke-free legislation period (before/after 1 July 2007 periods) and introduction of financial reward for recording of smoking information (before/after 1 April 2004) by including an interaction term between smoking status and the appropriate variable. Associations between the outcomes and time since smoking cessation were assessed using current and never smokers as reference groups. In sensitivity analyses: we compared imputed (including individuals with observed and imputed smoking status data, as in the primary analysis) and complete case (including only patients with recorded smoking status data (*n* = 1 413 749)] results, and examined associations separately by ignoring primary care recorded endpoints, restricting the analyses to fatal events and including first occurrence of each CVD endpoint irrespective of other earlier CVD presentation. Analyses were performed with R 3.0 and Stata 12.

### Ethical considerations

Approval was granted by the Independent Scientific Advisory Committee of the Medicines and Healthcare Products Regulatory Agency and the MINAP Academic Group. We registered the protocol at: [http://clinicaltrials.gov] (NCT01164371).

## Results

### Patient characteristics

A total of 1 937 360 subjects experienced 114 859 fatal or non-fatal CVD endpoints over 11.6 million person-years of follow-up (median 5.5 years per person). Of the total number of initial presentations of CVD events recorded, 14.1% were MI, 12.5% heart failure, 11.5% stable angina, 10.2% TIA, PAD 10.0%, ischaemic stroke 5.3%, unstable angina 4.9%, CA-SCD 2.9%, AAA 2.7%, intracerebral haemorrhage 2.1% and SAH 1.1%.

Of patients with recorded smoking status (1.4 million/1.9 million), 20.3% were active smokers (23.6% of men and 17.5% of women) and 16.2% ex-smokers (18.0% and 14.7%, respectively); 47% of current smokers and 49.1% of ex-smokers were women (Table 1). A description of smoking patterns of individuals included in the study by sex, age and birth cohort is shown in Appendix 1.4 (see Supplementary data available at *IJE* online). The proportion of ex-smokers and the sex differences in proportions of never smokers increased with higher baseline age from 13.9% in the 30–39 year group to 21.6% in those aged 80 years or more, and from 5.0% to 21.7%, respectively. Compared with former or never smokers, current smokers were younger, of white ethnicity and more socially deprived. Ex-smokers received statin and antihypertensive medication more frequently and diabetes was also more common among men.

**Table 1.** Baseline patient characteristics^a^ by smoking status in men and women

Characteristics	All with smoking data		Current smokers		Ex-smokers		Never smokers		Missing smoking status	
	Men	Women	Men	Women	Men	Women	Men	Women	Men	Women
	*N* = 647 400	*N* = 766 349	*N* = 153 074	*N* = 134 056	*N* = 116 630	*N* = 112 547	*N* = 377 696	*N* = 519 746	*N* = 310 929	*N* = 212 682
Age, years [SD]	46.0 (14.3)	47.9 (16.0)	42.9 (12.0)	43.5 (12.9)	51.0 (15.8)	48.9 (16.6)	45.7 (14.2)	48.9 (16.3)	45.4 (14.1)	50.9 (17.4)
Birth cohort (%)										
Before 1920	32 499 (5.0)	54 834 (7.2)	4760 (3.1)	4754 (3.6)	8103 (7.0)	7619 (6.8)	19 636 (5.2)	4261 (8.2)	17 852 (5.7)	24 572 (11.6)
1920–29	28 200 (4.4)	48 746 (6.4)	2601 (1.7)	3313 (2.5)	8592 (7.4)	8260 (7.3)	17 007 (4.5)	37 173 (7.2)	13 348 (4.3)	18 674 (8.8)
1930–39	52 612 (8.1)	69 166 (9.0)	7165 (4.7)	7408 (5.5)	14 295 (12.3)	10 560 (9.4)	31 152 (8.3)	51 198 (9.9)	23 785 (7.7)	20 358 (9.6)
1940–49	86 834 (13.4)	105 418 (13.8)	17 091 (11.2)	16 572 (12.4)	20 427 (17.5)	16 345 (14.5)	49 316 (13.1)	72 501 (14.0)	45 532 (14.6)	30 533 (14.4)
1950–80	447 255 (69.1)	488 185 (63.7)	121 457 (79.4)	102 009 (76.1)	65 213 (55.9)	69 763 (62.0)	260 585 (69.0)	316 413 (60.9)	210 412 (67.7)	118 545 (55.7)
Ethnicity (%)										
White	289 560 (89.1)	413 872 (89.8)	72 758 (89.3)	80 413 (95.1)	59 178 (92.1)	67 907 (95.0)	157 624 (87.8)	265 552 (87.1)	114 681 (93.6)	103 588 (94.1)
South Asian	12 033 (3.7)	14 525 (3.2)	2627 (3.2)	623 (0.7)	1521 (2.4)	653 (0.9)	7885 (4.4)	13 249 (4.4)	2069 (1.7)	1305 (1.2)
Black	11 025 (3.4)	15 106 (3.3)	2612 (3.2)	1451 (1.7)	1458 (2.3)	1060 (1.5)	6955 (3.9)	12 595 (4.1)	2950 (2.4)	2523 (2.3)
IMD, quintiles (%)										
Least deprived	136 810 (21.1)	161 224 (21.0)	21 199 (13.9)	17 769 (13.3)	25 083 (21.5)	23 818 (21.2)	90 528 (24.0)	119 637 (23.0)	54 798 (17.6)	36 329 (17.1)
Most deprived	124 319 (19.2)	141 960 (18.5)	44 276 (28.9)	38 513 (28.7)	20 028 (17.2)	19 460 (17·3)	60 015 (15.9)	83 987 (16.2)	71 212 (22.9)	49 294 (23.2)
Diabetes mellitus (%)	23 113 (3.6)	20 427 (2.7)	5054 (3.3)	3110 (2.3)	7092 (6.1)	3954 (3.5)	10 967 (2.9)	13 363 (2.6)	3809 (1.2)	2949 (1.4)
Hypertension (%)	185 656 (55.4)	241 812 (48.2)	41 033 (46.0)	36 265 (38.2)	45 959 (62.5)	39 162 (47.6)	98 664 (57.3)	166 385 (51.2)	39 221 (77.7)	48 904 (65.8)
BMI, kg/m^2^ (SD)	26.7 (4.5)	26.1 (5.7)	26.0 (4.6)	25.7 (5.7)	27.4 (4.5)	26.6 (5.8)	26.8 (4.4)	26.1 (5.7)	27.3 (5.0)	26.7 (6.0)
SBP, mmHg (SD)	133.0 (17.0)	127.4 (19.6)	130.2 (16.5)	124.0 (17.9)	134.8 (17.1)	127.7 (19.2)	132.8 (17.1)	128.4 (20.1)	137.1 (19.0)	130.9 (21.5)
DBP, mmHg (SD)	80.1 (10.0)	77.1 (10.2)	79.2 (10.1)	76.1 (10.2)	80.4 (9.8)	76.9 (10.1)	80.5 (9.9)	77.4 (10.2)	81.8 (10.3)	78.0 (10.7)
Total cholesterol, mmol/l (SD)	5.3 (1.1)	5.5 (1.2)	5.3 (1.2)	5.5 (1.2)	5.2 (1.1)	5.4 (1.2)	5.3 (1.1)	5.5 (1.2)	5.5 (1.1)	5.7 (1.2)
HDL cholesterol, mmol/l (SD)	1.3 (0.4)	1.5 (0.4)	1.2 (0.4)	1.5 (0.4)	1.3 (0.4)	1.6 (0.4)	1.3 (0.4)	1.6 (0.4)	1.3 (0.4)	1.6 (0.5)
Statin prescription (%)	26 282 (4.1)	24 402 (3.2)	6041 (4.0)	4166 (3.1)	9837 (8.4)	6102 (5.4)	10 404 (2.8)	14 134 (2.7)	2772 (0.9)	2238 (1.1)
Blood pressure-lowering medication (%)	101 063 (15.6)	157 040 (20.5)	19 075 (12.5)	22 073 (16.5)	27 358 (23.5)	25 992 (23.1)	54 630 (14.5)	108 975 (21.0)	25 397 (8.2)	34 731 (16.3)

BMI, body mass index; DBP, diastolic blood pressure; HDL high-density lipoprotein; IMD, index of multiple deprivation; SBP, systolic blood pressure.

^a^Missing data (%): ethnic group, 47.4%; BMI, 69.5%; blood pressure, 57.5%; total cholesterol, 91.4%; HDL cholesterol, 94.5%.

### Lifetime incidence of CVDs

**Figure 1.**
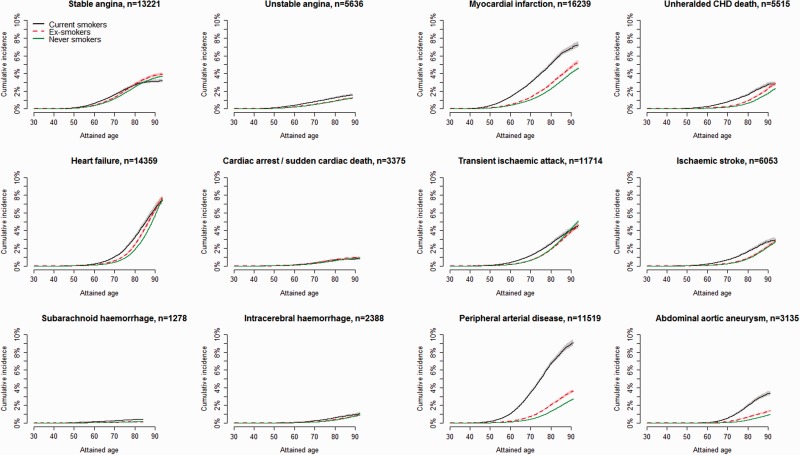
Lifetime cumulative incidence of 12 CVD phenotypes stratified by baseline smoking status. Shaded areas indicate confidence intervals.

The lifetime incidence of CVD amongst current, ex- and never smokers differed markedly across endpoints (Figure 1).The highest estimates were observed for PAD, occurring by age 90 years in 8.9% of current smokers and in 2.6% of never smokers, see Appendix 2.1 (available as Supplementary data at *IJE* online). Current smokers had an approximately 10-year earlier onset and higher and persisting incidence of MI, PAD and AAA through to older age. Smaller differences in age of onset and incidence were observed for other types of cerebrovascular phenotypes, unstable angina or heart failure. No differences according to smoking status were seen in lifetime risks for stable angina or CA-SCD.

### Current smoking and CVD

**Figure 2.**
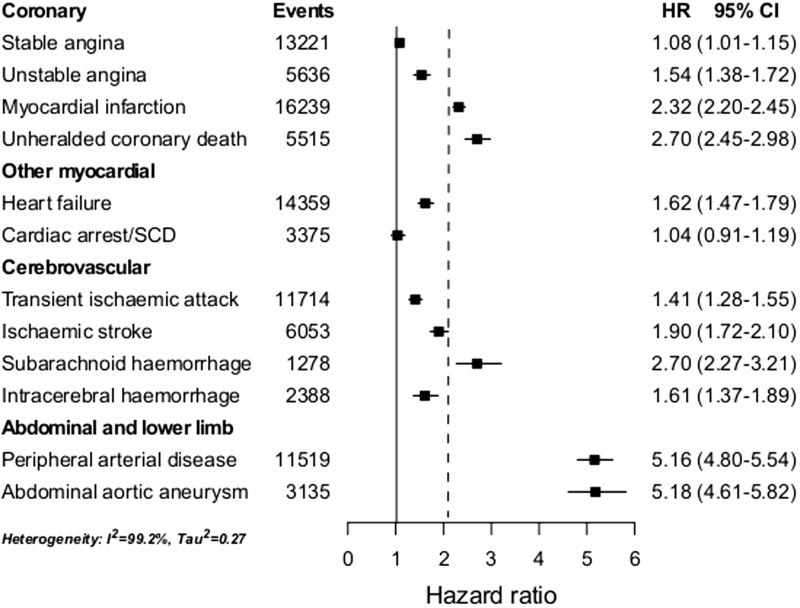
Age-adjusted hazard ratios of 12 CVDs comparing current vs never smokers. Cardiac arrest/SCD, cardiac arrest, ventricular fibrillation and sudden cardiac death; CI, confidence interval; HR, hazard ratio derived from Cox proportional hazard models with baseline hazard function stratified by sex and general practice and adjusted for baseline age (linear and quadratic terms); MI, myocardial infarction. The vertical dotted line indicates the HR of the composite cardiovascular disease endpoint.

Current smokers had increased hazard of most, but not all types of CVD (Figure 2). CA-SCD was not associated with smoking (adjusted HR = 1.04, 95% CI 0.91–1.19). Marked differences in the strength of associations were observed across endpoints (I^2 ^= 99.2%, τ^2 ^= 0.27), ranging from: weak with stable angina (adjusted HR = 1.08, 95% CI 1.01–1.15); moderate with TIA, unstable angina, intracerebral haemorrhage and heart failure (range 1.41 to 1.62); strong with ischaemic stroke and MI (adjusted HRs 1.90 and 2.32, respectively); and very strong with AAA, PAD, unheralded coronary death and SAH (range 2.70 to 5.18). Adjustment for other risk factors (see Appendix 2.2.1, available as Supplementary data at *IJE* online), comparison between complete case vs imputed data (see Appendix 2.2.2, available as Supplementary data at *IJE* online), restricting data sources (see Appendix 2.2.3, available as Supplementary data at *IJE* online) and analysing endpoints defined as first events (irrespective of other earlier CVD presentation; see Appendix 2.2.4, available as Supplementary data at *IJE* online) made little if any difference to our finding of highly heterogeneous associations. Adjusted HRs for STEMI and NSTEMI were 2.60 (95% CI 2.35–2.88) and 2.30 (95% CI 2.07–2.56), respectively.

### Interactions with sex

**Figure 3.**
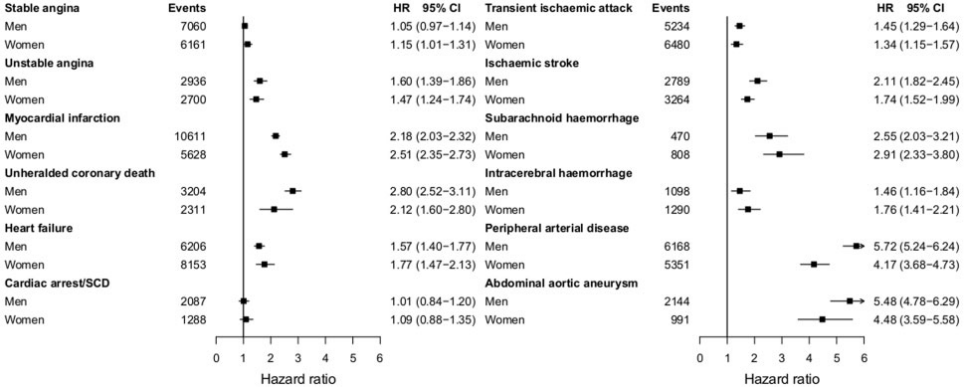
Age-adjusted hazard ratios of 12 CVDs comparing current vs never smokers in men and women. Cardiac arrest/SCD, cardiac arrest, ventricular fibrillation and sudden cardiac death; CI, confidence interval; HR, age-adjusted hazard ratio from Cox proportional hazard models with baseline hazard function stratified by general practice and adjusted for baseline age (linear and quadratic terms). *P-value for interaction ≤0.05 (*P* = 0.05 for myocardial infarction and *P* = 0.0005 for peripheral arterial disease).

The relationship between current smoking and PAD was stronger in men than in women (adjusted HR = 5.72, 95% CI 5.24–6.24; compared with 4.17, 95% CI 3.68–4.73; interaction *P*-value 0.0005; see Figure 3). In contrast, current smoking showed stronger associations among women than in men for MI (adjusted HR = 2.51, 95% CI 2.35–2.73 vs 2.18, 95% CI 2.03–2.32; interaction *P*-value 0.05).

### Population-attributable fractions

**Figure 4.**
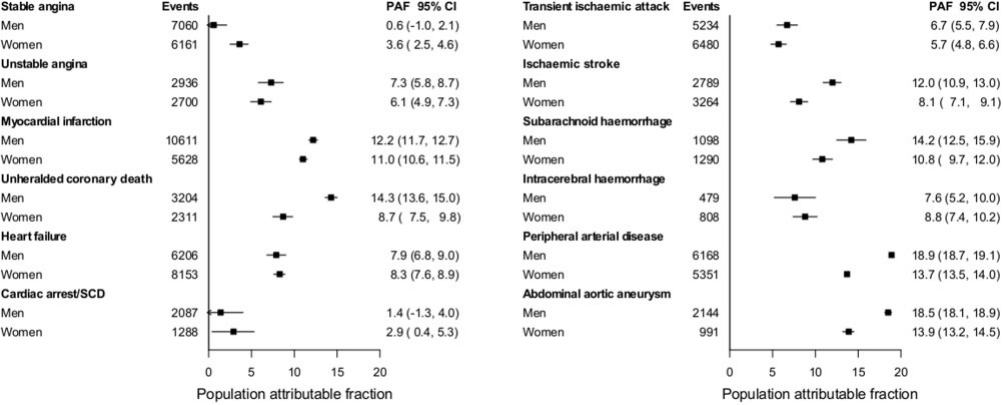
Population-attributable fractions (%) for 12 CVDs associated with current smoking in men and women. Cardiac arrest/SCD, cardiac arrest, ventricular fibrillation and sudden cardiac death; CI, confidence interval; CVD, cardiovascular diseases; PAF, population-attributable fraction derived from Cox proportional hazard models with baseline hazard function stratified by sex and general practice and adjusted for baseline age (linear and quadratic terms).

Overall PAF for combined CVDs for current smoking was 10.6% (95% CI 10.5%-10.8%). This summary estimate masked a wide range, from 2.2% for stable angina to 16.3% for AAA and PAD (see Appendix 2.3, available as Supplementary data at *IJE* online). PAF estimates were markedly lower for women than men for unheralded coronary death (8.7% vs 14.3%), ischaemic stroke (8.1% vs 12.0%), PAD (13.7% vs 18.9%) and AAA (13.9% vs 18.5%; see Figure 4).

### Associations among high-risk groups

Age modified the smoking associations in disease-specific patterns. For angina and CA-SCD, higher HRs among current smokers were confined to the younger age groups (adjusted HR = 1.48, 95% CI 1.15–1.90, interaction *P*-value <0.0001; and 1.40, 95% CI 1.03–1.90, interaction *P*-value 0.05, respectively, among 30–39-year-olds). For other diseases, although the HR tended to decline with age, estimates remained elevated into the 8th and 9th decades of life (Appendices 2.4.1, 2.4.2 and 2.4.3, available as Supplementary data at *IJE* online).

Relationships between current smoking and CVD phenotypes were weaker in individuals with diabetes or hypertension. Weaker associations in smoking among people with diabetes compared with those without diabetes were found for unstable angina, PAD and MI (percentage decreases were 43.4%, 42.4% and 28.0%, respectively; interaction *P*-values 0.04, <0.0001 and 0.001, respectively; see Appendix 2.4.4, available as Supplementary data at *IJE* online). Greater differences between patients with and without hypertension were found for PAD (23.3% percentage decrease, interaction *P*-value 0.004; see Appendix 2.4.5, available as Supplementary data at *IJE* online).

### Smoking cessation

**Figure 5.**
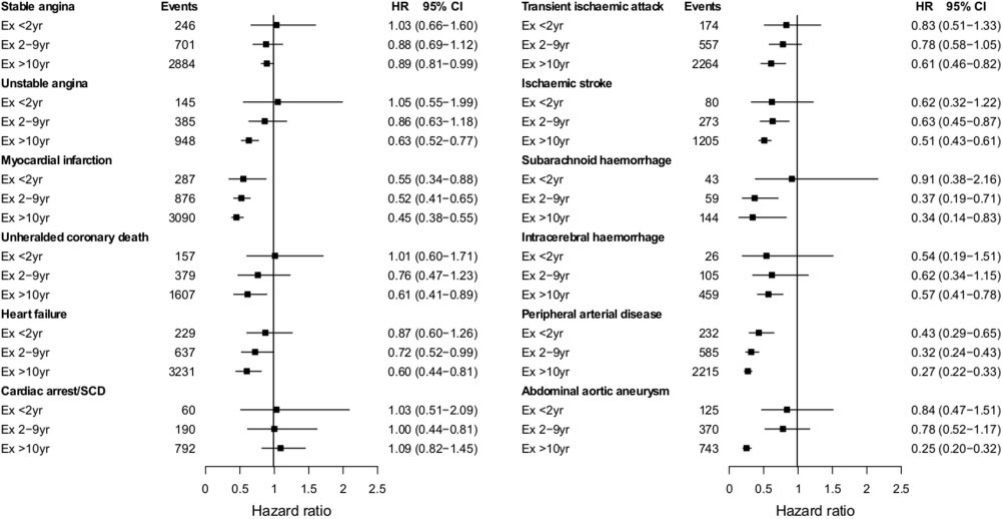
Age-adjusted hazard ratios of 12 CVDs for duration of smoking cessation vs current smokers. Cardiac arrest/SCD, cardiac arrest, ventricular fibrillation and sudden cardiac death; CI, confidence interval; HR, hazard ratio derived from Cox proportional hazard models with baseline hazard function stratified by sex and general practice and adjusted for baseline age (linear and quadratic terms).

The hazard of all CVDs (except CA-SCD) gradually decreased with time after smoking cessation, with hazard ratios ranging between 0.43 and 1.03 for the first 2 years after quitting, and between 0.25 and 1.09 for the period after 10 years of quitting, compared with current smokers (Figure 5). Although individuals generally achieved the risk level of never smokers after 10 years of quitting (see Appendix 2.5, available as Supplementary data at *IJE* online), estimates for PAD (HR = 1.36, 95% CI 1.11–1.67) and AAA (HR = 1.47, 95% CI 1.10–1.95) in men, and unheralded coronary death (HR = 2.74, 95% CI 1.36–5.51) in women remained increased.

### Public health interventions

Associations with smoking status were similar in the periods before and after the introduction of two public health interventions on smoking, the financial reward for collection of smoking data (see Appendix 2.6.1, available as Supplementary data at *IJE* online) and the smoking ban in England (Appendix 2.6.2, available as Supplementary data at *IJE* online).

### Discriminative ability of smoking status

**Figure 6.**
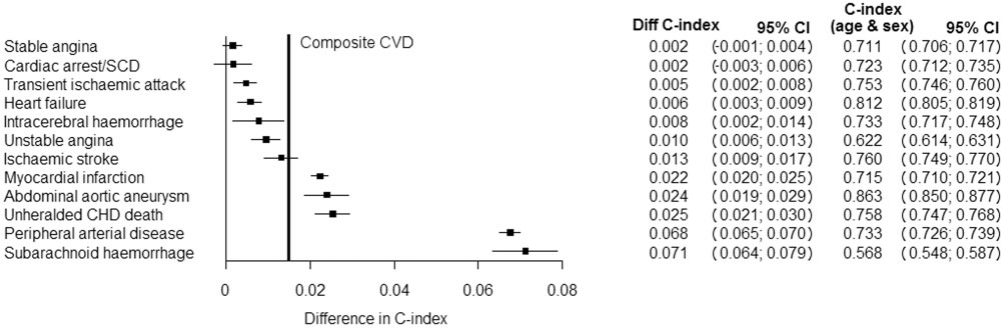
Increment in c-index associated with inclusion of smoking status in CVD phenotype-specific models containing sex and age. The vertical bold line indicates the increment in the c-index estimate for the CVD composite (increment index = 0.016, 95% CI 0.015-0.017), compared with a sex- and age-adjusted model with c-index = 0.73 (95% CI 0.72-0.73). Cardiac arrest/SCD, cardiac arrest, ventricular fibrillation and sudden cardiac death; CHD, coronary heart disease; CI, confidence interval; CVD, cardiovascular diseases.

The c-index increment when smoking coefficients were included in risk prediction models adjusted for age and sex varied from 0.2% for stable angina and CA-SCD to 7.1% for SAH, compared with an increment of 1.6% for the CVD composite (see Figure 6).

## Discussion

This population-based cohort analysis of contemporary electronic health records (1997–2010) from more than 1.9 million adults with more than 100 000 initial presentations of non-fatal and fatal CVDs showed that current smoking has highly heterogeneous associations with different types of disease. Estimates ranged from none for CA-SCD, through weak for stable angina, to very strong for AAA and PAD. Our findings point to underlying differences in disease aetiology, and have implications for risk prediction in clinical practice.

To our knowledge, we are the first to report the lifetime risks of different CVDs according to smoking status. Previous reported estimates of lifetime risks were calculated for aggregated risk factors and aggregated endpoints.^13^ Current guidelines recommend managing CVDs as a single family of related diseases,^24^ but our findings suggest important differences. The disease with the highest absolute lifetime risk among current smokers is PAD (9% risk by age 90 years, compared with 3% risk among never smokers), a disease that is considerably less studied than MI. Evaluating lifetime risks may help in counselling patients and in starting preventive measures at an appropriately early age.

The heterogeneous association we observed between current smoking and 12 different acute and chronic CVDs has not previously been reported. This is because previous studies have not collected data on this range of clinical phenotypes, or have lacked statistical power. Nonetheless, our findings are consistent with previously reported associations with a limited number of diseases,^25–28^ with stronger associations found for MI than for stable or unstable angina,^29^^,^^30^ for unstable than for stable angina^31^ and for SAH than for other types of stroke.^32^^,^^33^ Stronger associations were also seen in younger than in older individuals.^27^^,^^33^

The heterogeneity in estimates we observed between smoking and different manifestations of CVD likely reflects differences in pathophysiological mechanisms. The absence of association with CA-SCD, for instance, contrasts with the strong association with acute MI and is consistent with mechanistic differences in the risk of these disorders reflected in the observation that the heart rate profile during exercise associates with CA-SCD but not with MI.^34^ Other investigators have reported an increased risk of SCD among current smokers,^35^^,^^36^ although this apparent contradiction may be because they did not focus on SCD as an initial presentation of CVD. The hazard of all CVDs decreased after smoking cessation, with faster decreases observed for MI and PAD (by 50–57% within 2 years of quitting), and individuals generally achieving the level of never smokers 10 years after quitting. Our findings are consistent with reports from previous studies of MI^27^ and stroke^37^^,^^38^ among ex-smokers. The rapid decreases in risk of MI and PAD after smoking cessation suggest that the initial presentation of these two CVDs is likely to be triggered by acute effects of smoke exposure acting on the vascular system. This finding is consistent with the observation of sharp declines in rates of acute MI early after the introduction of smoke-free legislation, and effects increasing over time since implementation.^3^

We report sex differences in public health impact which might arise if no one smoked. In a meta-analysis of 75 cohorts and 2.4 million people recruited in 1956–2000, women smokers had a 25% higher risk of aggregates of CHD than men smokers, with differences found only in the 60–69-year-old group.^6^ These results are consistent with our estimates for MI but contradict those for PAD, an endpoint not reported in many studies.

PAFs have not previously been compared across the initial presentation of these 12 CVDs. Assuming a causal relationship, abolishing smoking would prevent a higher proportion of some diseases (e.g. AAA, PAD) than others (e.g. CA-SCD, angina). Moreover, for four of the diseases studied (unheralded coronary death, ischaemic stroke, SAH and intracerebral haemorrhage), this proportion would be substantially higher among men.

We found strong evidence that incorporating a single generic effect of smoking in widely used risk prediction models for CVD aggregates will overestimate the risk of some diseases (e.g. heart failure or CA-SCD) and underestimate the risk of others (e.g. PAD). Differences in the discriminative capacity of smoking across types of CVD ranged from 0.2% for stable angina to 7% for SAH. Further, we show that age (strongly), and to a lesser extent diabetes and hypertension, modify associations between smoking and CVDs. All these findings have implications for ‘precision medicine’ in targeting primary prevention strategies. Clinicians can now provide patients with greater clarity about the heterogeneous hazards of smoking across a broad range of CVD presentations, and greater accuracy of risk prediction for specific types of CVD. In an ageing population, patients need to be informed how smoking affects lifetime risks of different CVDs, for example lifetime risks of PAD and AAA are no lower than risks of MI. Further, younger people confident that CVD risk will disappear after quitting should know that this does not apply across all CVDs; and, in those who manage to quit risks of PAD, AAA and unheralded coronary death remain increased even after 10 years. Our findings also emphasise the importance of defining specific cardiovascular endpoints, and of understanding the composition of aggregate endpoints used in observational studies, clinical trials and cost-impact evaluations of interventions to promote smoking cessation. They also stress the need to report and consider CVD-specific information when assessing heterogeneity in meta-analyses and in design and interpretation of gene-environment interaction studies.^39^

Two public health interventions were implemented during the study period. In 2004, general practitioners began to be financially rewarded for recording information on smoking status and this may have triggered risk-lowering interventions among smokers. In 2007, the public smoking ban was introduced in England and this might have lowered passive exposure among non-smokers. Whereas there is good evidence that the smoking ban was associated with reduced rates of MI in the UK and elsewhere,^3^ we did not find strong evidence that either of these initiatives changed the associations of smoking status across a wide range of CVDs in the present study.

Smoking status is among the most important pieces of information that a healthcare professional can record.^40^ Our study highlights the prospective validity of such a measure in research, and points to the need for quality-of-care initiatives to improve recording. About a quarter of patients (27%) had no clinical record of smoking status and also details were lacking on age at initiation, patterns and duration of smoking, amount of cigarettes smoked and second-hand smoke exposure. More detailed measures of smoking are likely to show stronger relationships with disease. For example, among women selected from a national breast cancer screening programme,^5^ the amount of cigarettes smoked was associated with stronger relative risks of cause-specific mortality than current smoking. Our findings support previous calls, embodied in a National Institute of Health and Care Excellence (NICE) quality framework,^41^ for healthcare professionals to regularly question patients as a tool to encourage discussion, review options currently available to support smoking cessation and monitor progress made; and to record detailed information about smoking behaviour for research purposes.

As research and meaningful re-use of EHRs is encouraged worldwide, ours is among the first large-scale cohorts to demonstrate the potential of using linked clinically recorded health information on health-related behaviour for testing new aetiological hypotheses. The large population-based sample and number of CVD endpoints analysed, the longitudinal design allowing differentiation between incident initial CVD presentations and progression of disease, and the availability of information about a wide range of common cardiovascular phenotypes are some of the strengths of our study. There is evidence of the completeness and validity of diagnostic coding of the original data sources,^42–44^ and the use of linked individual patient data from four different sources for the identification of endpoints minimized the likelihood of outcome status misclassification.^42^^,^^45^

Several limitations are to be considered when interpreting our findings. First, data for smoking and risk factors were missing for some patients, but findings based on multiply imputed data and complete case analyses were consistent. Second, information on detailed smoking habits (e.g. patterns, duration and amount of cigarettes smoked) was unavailable and residual confounding cannot be completely excluded. However, adjustment for the main known or suspected cardiovascular risk factors, for medication use and for patient birth cohort (data not shown) had little effect on almost all the estimates. Third, smoking status was self-reported by patients during consultations with their general practitioner and might have been misreported by some. Smoking status might also have changed over time, and this could have resulted in underestimation of associations (e.g. for CA-SCD) and overestimation of the length of time required to achieve the risk levels of never smokers after smoking cessation. Fourth, to define CVDs we used data from four different EHR sources, each of which has its own error. However, smoking associations were robust to exclusion of primary care cases or non-fatal cases; and we^42^ and others^46^ have provided evidence of the validity of using linkages for endpoint follow-up. Fifth, we were unable to resolve disease subtypes including systolic or diastolic heart failure, which might mask an even greater degree of heterogeneity. Finally, we cannot exclude that some associations might have resulted from multiple testing, and caution is required to interpret reported confidence intervals.

## Conclusion

The highly heterogeneous associations of smoking across different types of cardiovascular phenotypes have important implications for research, clinical screening and risk prediction. They suggest the need to move away from aggregate to disease-specific risk models that are more informative for clinicians and policymakers in developing and implementing strategies for the prevention of CVD.

## Supplementary Data

Supplementary data are available at *IJE* online.

## Funding

This work was supported by grants from: the Wellcome Trust [WT 086091/Z/08/Z]; the UK National Institute for Health Research (RP-PG-0407-10314); and by awards establishing the Farr Institute of Health Informatics Research at UCL Partners from the Medical Research Council (MR/K006584/1), in partnership with Arthritis Research UK; the British Heart Foundation; Cancer Research UK; the Economic and Social Research Council; the Engineering and Physical Sciences Research Council; the National Institute of Health Research; the National Institute for Social Care and Health Research (Welsh Assembly Government); the Chief Scientific Office (Scottish Government Health Directorates); and the Wellcome Trust. J.G. was funded by an NIHR Doctoral Fellowship [DRF-2009-02-50]; L.S. is supported by a Wellcome Trust Senior Research Fellowship in Clinical Science; A.S. is supported by a Wellcome Trust Clinical Research Training Fellowship [0938/30/Z/10/Z]; A.T. is supported by Barts and the London Cardiovascular Biomedical Research Unit funded by the National Institute for Health Research; and R.W. by Cancer Research UK. The views and opinions expressed therein are those of the authors and do not necessarily reflect those of the NIHR PHR Programme or the Department of Health.

The authors declare that the funding sources had no role in the conduct, analysis, interpretation or writing of this manuscript.

## Contributors

M.P.R. participated in the study design, protocol development, coding algorithms, conduct of the literature review, study implementation and coordination, performed the data analysis and wrote the manuscript. J.G. participated in the study design, coding algorithms and protocol development. A.S. participated in the study design, coding algorithms and protocol development. E.R. participated in the study design, data analysis and report writing. S.D. participated in the implementation of the coding algorithms, preparation of the dataset and documentation. R.W. participated in the critical revision of the manuscript. L.S. participated in the protocol development, verification of code lists, critical revision of the manuscript and secured grant. A.T. participated in the protocol development, verification of code lists and secured grant. H.H. participated in the study design, protocol development, verification of code lists, report writing and secured grant.

**Conflict of interest:** The authors declare no conflicts of interest. R.W. has undertaken research and consultancy for the following companies that develop and manufacture smoking cessation medications: Pfizer, GSK, J & J and Sanofi-Aventis. He is trustee of the stop-smoking charity, QUIT, and honorary co-director of the UK’s National Centre for Smoking Cessation and Training.

## Supplementary Material

Supplementary Data
