# Supplementary material for: Heterogeneous associations between smoking and a wide range of initial presentations of cardiovascular disease in 1 937 360 people in England: lifetime risks and implications for risk prediction
Source: Int J Epidemiol. 2014 Nov 20;44(1):129–41. doi: 10.1093/ije/dyu218 (PMC4339760; doi:10.1093/ije/dyu218)
Supplement: Supplementary Data [file supp_44_1_129__index.html]

Heterogeneous associations between smoking and a wide range of initial presentations of cardiovascular disease in 1 937 360 people in England: lifetime risks and implications for risk prediction — Heterogeneous associations between smoking and a wide range of initial presentations of cardiovascular disease in 1 937 360 people in England: lifetime risks and implications for risk prediction — Supplementary Data 

# Heterogeneous associations between smoking and a wide range of initial presentations of cardiovascular disease in 1 937 360 people in England: lifetime risks and implications for risk prediction

## Supplementary Data

files

**Files in this Data Supplement:**

- Supplementary Data - docx file
