# Supplementary material for: Heterogeneous associations between smoking and a wide range of initial presentations of cardiovascular disease in 1 937 360 people in England: lifetime risks and implications for risk prediction
Source: Int J Epidemiol. 2014 Nov 20;44(1):129–41. doi: 10.1093/ije/dyu218 (PMC4339760; doi:10.1093/ije/dyu218)
Supplement: Supplementary Data [file supp_dyu218_ije-2014-03-0289-File002.docx]

**SUPPLEMENTAL MATERIAL**

**CONTENT**

**1. SUPPLEMENTAL METHODS**

**1.1. CALIBER program: study data sources**

**1.2 Study flow diagram**

**1.3 CALIBER web links to cardiovascular endpoint definitions**

**1.4 Smoking patterns in individuals included in the study**

**SUPPLEMENTAL RESULTS**

**2.1 Lifetime risks of twelve cardiovascular diseases associated with smoking status at different index ages up to age 90**

**2.2 Further adjustment and sensitivity analyses**

2.2.1 Effect of adjusting for baseline established cardiovascular risk factors on age-adjusted hazard ratios for the association between current vs. never smokers and twelve cardiovascular diseases

2.2.2 Age-adjusted hazard ratios for the association between current vs. never smoking and twelve cardiovascular diseases from observed (complete case analysis) or imputed analyses

2.2.3 Age-adjusted hazard ratios for the association between current vs. never smoking and twelve cardiovascular diseases by source of endpoint

2.2.4 Age-adjusted hazard ratios for the association between current vs. never smoking and twelve cardiovascular diseases with endpoints defined as initial presentation or first event

**2.3 Population attributable fractions with 95% confidence intervals for the association between current and former smoking and twelve cardiovascular diseases stratified by sex, based on estimates from models adjusted for age and with baseline hazard function stratified by sex and practice**

**2.4 Interactions with sex, age, diabetes and hypertension**

2.4.1 Hazard ratios for the association between current vs. never smokers and twelve cardiovascular diseases by baseline age group in men and women

2.4.2 Hazard ratios for the association between current smoking and twelve cardiovascular diseases by baseline age group in men

2.4.3 Hazard ratios for the association between current smoking and twelve cardiovascular diseases by baseline age group in women

2.4.4 Age-adjusted hazard ratios for the association between current vs. never smoking and twelve cardiovascular diseases in patients with or without diabetes

2.4.5 Age-adjusted hazard ratios for the association between current vs. never smoking and twelve cardiovascular diseases in patients with or without hypertension

**2.5 Age-adjusted hazard ratios for the association between duration of smoking cessation and twelve cardiovascular diseases (ref. never smokers)**

**2.6 Public health interventions**

2.6.1 Age-adjusted hazard ratios for the association between current vs. never smoking and twelve cardiovascular diseases in the period before and after the introduction of financial reward for recording of smoking data (April 2004)

2.6.2 Age-adjusted hazard ratios for the association between current vs. never smoking and twelve cardiovascular diseases in the period before and after implementation of the smoke-free legislation in England (January 2007)

**1. SUPPLEMENTAL METHODS**

**1.1 CALIBER program: study data sources**

The CALIBER (Cardiovascular disease research using Linked Bespoke studies and Electronic Records) program was conceived-gain understanding of the aetiology and prognosis of specific coronary phenotypes[^1^](#_ENREF_10). Electronic medical records from patients registered in 225 family practices were linked across four data sources: the Clinical Practice Research Datalink (CPRD)^2^; the Myocardial Ischaemia National Audit Project registry (MINAP)^3^; Hospital Episodes Statistics (HES); and the Office of National Statistics (ONS). CPRD provides primary care data on health behaviours, diagnoses, investigations, procedures and prescriptions; and its accuracy and completeness are regularly audited. MINAP is a national registry of patients hospitalised with acute coronary syndromes in England and Wales. HES provides information on all hospital admissions and ONS cause-specific mortality records for all deaths in England and Wales. Information is coded using the hierarchical clinical coding schemes (Read[^4^](#_ENREF_11), the International Statistical Classification of Diseases and Health Related Problems, 10th revision[^5^](#_ENREF_12), and Office of the Population Censuses and Surveys Classification of Interventions and Procedures[^6^](#_ENREF_13)).

**Reference List**

1. Denaxas SC, George J, Herrett E, Shah AD, Kalra D, Hingorani AD, et al. Data resource profile: cardiovascular disease research using linked bespoke studies and electronic health records (CALIBER). Int J Epidemiol. 2012; **41**: 1625-38.
2. Walley T, Mantgani A. The UK General Practice Research Database. Lancet. 1997; **350**: 1097-1099.
3. Herrett E, Smeeth L, Walker L, Weston C. The Myocardial Ischaemia National Audit Project (MINAP). Heart. 2010; **96**(16):1264-7.
4. Chisholm J. The Read clinical classification. BMJ. 1990; **300**: 1092.
5. International Classification of Diseases and Related Health Problems, Tenth Revision (ICD-10). Vols 1-3. Geneva, World Health Organization, 1992-2000. Fourth Edition.
6. OPCS-4 Classification - NHS Connecting for Health; 2013.

**1.2 Study flow diagram**

5 372 790 CALIBER patients

4 703 682 patients met research quality standards

1 937 360 patients included

**Patients excluded:**

- Missing sex = 135
- Age <30 years = 1 858 924
- <1 year follow-up prior to study entry = 709 006
- History of CVD prior to entry date = 39 018
- Pregnant within 6 months of eligibility date = 159 239

669 108 suboptimal research quality data

**1.3 CALIBER web links to cardiovascular endpoint definitions**

| **Endpoint** | **Web link** |
| --- | --- |
| Stable angina | <https://www.caliberresearch.org/portal/show/phenotype_sa> |
| Unstable angina | <https://www.caliberresearch.org/portal/show/phenotype_ua> |
| Myocardial infarction | <https://www.caliberresearch.org/portal/show/phenotype_mi> |
| Unheralded coronary death | <https://www.caliberresearch.org/portal/show/phenotype_chd> (only fatal events) |
| Heart failure | <https://www.caliberresearch.org/portal/show/phenotype_hf> |
| Sudden cardiac death, cardiac arrest and ventricular arrhythmia | <https://www.caliberresearch.org/portal/show/phenotype_scd> |
| Transient ischemic attack | <https://www.caliberresearch.org/portal/show/phenotype_tia> |
| Subarachnoid haemorrhage | <https://www.caliberresearch.org/portal/show/phenotype_stroke_subarachnoid> |
| Ischaemic stroke | <https://www.caliberresearch.org/portal/show/phenotype_stroke_ischaemic> |
| Intracerebral haemorrhage | <https://www.caliberresearch.org/portal/show/phenotype_stroke_intracerebral_haem> |
| Peripheral arterial disease | <https://www.caliberresearch.org/portal/show/phenotype_pad> |
| Abdominal aortic aneurysm | <https://www.caliberresearch.org/portal/show/phenotype_aaa> |

**1.4 Smoking patterns in individuals included in the study**

**A) Percentage of individuals stratified by age group at baseline B) Percentage of individuals stratified by sex and age group at baseline**

**Note:** Continuous lines link estimates from men and dashed lines estimates from women.

**C) Percentage of individuals stratified by sex and birth cohort**

**2. SUPPLEMENTAL RESULTS**

**2.1 Lifetime risks of twelve cardiovascular diseases associated with smoking status at different ages up to age 90**

| **Age (years)** | **Current smokers** | **Ex-smokers** | **Never smokers** |
| --- | --- | --- | --- |
|  | **Percentage lifetime risk (95% CI)** | **Percentage lifetime risk (95% CI)** | **Percentage lifetime risk (95% CI)** |
| **Stable angina** |  |  |  |
| 40 | 0 (0-0) | 0 (0-0) | 0 (0-0) |
| 50 | 0.1 (0.1-0.2) | 0.1 (0.1-0.1) | 0.1 (0.1-0.1) |
| 60 | 0.6 (0.6-0.7) | 0.4 (0.4-0.5) | 0.4 (0.4-0.4) |
| 70 | 1.6 (1.5-1.7) | 1.3 (1.2-1.4) | 1.2 (1.1-1.2) |
| 80 | 2.7 (2.6-2.9) | 2.8 (2.6-2.9) | 2.5 (2.4-2.5) |
| 90 | 3.1 (3.0-3.3) | 3.8 (3.6-4.0) | 3.5 (3.4-3.6) |
| **Unstable angina** |  |  |  |
| 40 | 0 (0-0) | 0 (0-0) | 0 (0-0) |
| 50 | 0.1 (0.1-0.2) | 0.1 (0.1-0.1) | 0.1 (0.1-0.1) |
| 60 | 0.4 (0.4-0.4) | 0.2 (0.2-0.2) | 0.2 (0.2-0.2) |
| 70 | 0.8 (0.8-0.9) | 0.5 (0.5-0.5) | 0.5 (0.5-0.5) |
| 80 | 1.3 (1.2-1.3) | 0.9 (0.8-0.9) | 0.9 (0.8-0.9) |
| 90 | 1.6 (1.5-1.7) | 1.3 (1.2-1.4) | 1.3 (1.2-1.3) |
| **Myocardial infarction** |  |  |  |
| 40 | 0 (0-0.1) | 0 (0-0) | 0 (0-0) |
| 50 | 0.4 (0.4-0.4) | 0.1 (0.1-0.1) | 0.1 (0.1-0.1) |
| 60 | 1.4 (1.3-1.4) | 0.5 (0.4-0.5) | 0.4 (0.4-0.4) |
| 70 | 3.0 (2.9-3.1) | 1.3 (1.2-1.4) | 1.0 (1.0-1.1) |
| 80 | 5.1 (4.9-5.3) | 2.8 (2.7-2.9) | 2.3 (2.2-2.3) |
| 90 | 6.8 (6.5-7.1) | 2.8 (2.7-2.9) | 4.1 (4.0-4.2) |
| **Unheralded coronary death** |  |  |  |
| 40 | 0 (0-0) | 0 (0-0) | 0 (0-0) |
| 50 | 0.1 (0.1-0.1) | 0 (0-0) | 0 (0-0) |
| 60 | 0.3 (0.2-0.3) | 0.1 (0.1-0.1) | 0.1 (0.1-0.1) |
| 70 | 0.8 (0.8-0.9) | 0.3 (0.2-0.3) | 0.2 (0.2-0.2) |
| 80 | 1.6 (1.5-1.7) | 0.9 (0.8-1.0) | 0.7 (0.6-0.7) |
| 90 | 2.8 (2.5-3.0) | 2.3 (2.1-2.5) | 1.7 (1.7-1.8) |
| **Cardiac arrest/SCD** |  |  |  |
| 40 | 0 (0-0) | 0 (0-0) | 0 (0-0) |
| 50 | 0 (0-0.1) | 0 (0-0) | 0 (0-0) |
| 60 | 0.2 (0.1-0.2) | 0.1 (0.1-0.1) | 0.1 (0.1-0.1) |
| 70 | 0.4 (0.3-0.4) | 0.3 (0.3-0.4) | 0.3 (0.3-0.3) |
| 80 | 0.7 (0.6-0.8) | 0.7 (0.7-0.8) | 0.6 (0.6-0.6) |
| 90 | 0.8 (0.7-0.9) | 0.9 (0.9-1.0) | 0.9 (0.8-0.9) |
| **Heart failure** |  |  |  |
| 40 | 0 (0-0) | 0 (0-0) | 0 (0-0) |
| 50 | 0 (0-0.1) | 0 (0-0) | 0 (0-0) |
| 60 | 0.3 (0.2-0.3) | 0.1 (0.1-0.1) | 0.1 (0.1-0.1) |
| 70 | 1.0 (1.0-1.1) | 0.6 (0.5-0.6) | 0.4 (0.4-0.5) |
| 80 | 3.2 (3.1-3.4) | 2.4 (2.3-2.5) | 1.9 (1.8-1.9) |
| 90 | 6.5 (6.1-6.8) | 6.2 (6.0-6.5) | 5.7 (5.6-5.8) |
| **Transient ischaemic attack** |  |  |  |
| 40 | 0 (0-0) | 0 (0-0) | 0 (0-0) |
| 50 | 0.1 (0.1-0.1) | 0 (0-0) | 0 (0-0) |
| 60 | 0.4 (0.4-0.4) | 0.2 (0.2-0.2) | 0.2 (0.2-0.2) |
| 70 | 1.2 (1.1-1.3) | 0.6 (0.6-0.7) | 0.6 (0.6-0.7) |
| 80 | 2.5 (2.4-2.7) | 1.9 (1.8-2.0) | 1.9 (1.9-2.0) |
| 90 | 4.1 (3.8-4.3) | 3.9 (3.7-4.1) | 4.2 (4.1-4.3) |
| **Ischaemic stroke** |  |  |  |
| 40 | 0 (0-0) | 0 (0-0) | 0 (0-0) |
| 50 | 0.1 (0-0.1) | 0 (0-0) | 0 (0-0) |
| 60 | 0·2 (0.2-0.3) | 0.1 (0.1-0.1) | 0.1 (0.1-0.1) |
| 70 | 0.7 (0.6-0.8) | 0.3 (0.2-0.3) | 0.3 (0.2-0.3) |
| 80 | 1.6 (1.5-1.8) | 0.9 (0.8-1.0) | 0.8 (0.8-0.9) |
| 90 | 2.7 (2.5-3.0) | 2.2 (2.1-2.4) | 2.2 (2.1-2.2) |
| **Subarachnoid haemorrhage** |  |  |  |
| 40 | 0 (0-0) | 0 (0-0) | 0 (0-0) |
| 50 | 0.1 (0.1-0.1) | 0 (0-0) | 0 (0-0) |
| 60 | 0.2 (0.1-0.2) | 0.1 (0-0.1) | 0.1 (0-0.1) |
| 70 | 0.3 (0.2-0.3) | 0.1 (0.1-0.1) | 0.1 (0.1-0.1) |
| 80 | 0.4 (0.4-0.5) | 0.1 (0.1-0.2) | 0.2 (0.1-0.2) |
| 90 | 0.4 (0.4-0.5) | 0.2 (0.2-0.2) | 0.2 (0.2-0.2) |
| **Intracerebral haemorrhage** |  |  |  |
| 40 | 0 (0-0) | 0 (0-0) | 0 (0-0) |
| 50 | 0 (0-0) | 0 (0-0) | 0 (0-0) |
| 60 | 0.1 (0.1-0.1) | 0 (0-0.1) | 0 (0-0) |
| 70 | 0.3 (0.2-0.3) | 0.1 (0.1-0.2) | 0.1 (0.1-0.1) |
| 80 | 0.6 (0.5-0.7) | 0.4 (0.3-0.4) | 0.3 (0.3-0.4) |
| 90 | 1.0 (0.8-1.1) | 0.8 (0.7-0.9) | 0.8 (0.7-0.9) |
| **Peripheral arterial disease** |  |  |  |
| 40 | 0 (0-0) | 0 (0-0) | 0 (0-0) |
| 50 | 0 (0-0) | 0 (0-0) | 0 (0-0) |
| 60 | 1.1 (1.0-1.1) | 0.2 (0.2-0.2) | 0.1 (0.1-0.1) |
| 70 | 3.5 (3.3-3.6) | 0.7 (0.7-0.8) | 0.4 (0.4-0.5) |
| 80 | 6.7 (6.5-7.0) | 2.0 (1.9-2.1) | 1.3 (1.3-1.4) |
| 90 | 8.9 (8.6-9.3) | 3.5 (3.3-3.7) | 2.6 (2.5-2.7) |
| **Abdominal aortic aneurysm** |  |  |  |
| 40 | 0 (0-0) | 0 (0-0) | 0 (0-0) |
| 50 | 0 (0-0) | 0 (0-0) | 0 (0-0) |
| 60 | 0.1 (0.1-0.1) | 0 (0-0) | 0 (0-0) |
| 70 | 0.6 (0.5-0.6) | 0.2 (0.1-0.2) | 0.1 (0.1-0.1) |
| 80 | 2.0 (1.8-2.1) | 0.7 (0.6-0.7) | 0.4 (0.3-0.4) |
| 90 | 3.3 (3.1-3.6) | 1.3 (1.2-1.4) | 0.9 (0.8-1.0) |

Note: Cardiac arrest-SCD, cardiac arrest, ventricular fibrillation and sudden cardiac death; CI, confidence interval. Lifetime risk estimates expressed as percentages take into account competing risks.

**2.2 Further adjustment and sensitivity analyses**

2.2.1 Effect of adjusting for baseline established cardiovascular risk factors and for medication use on age-adjusted hazard ratios for the association between current vs. never smokers and twelve cardiovascular diseases


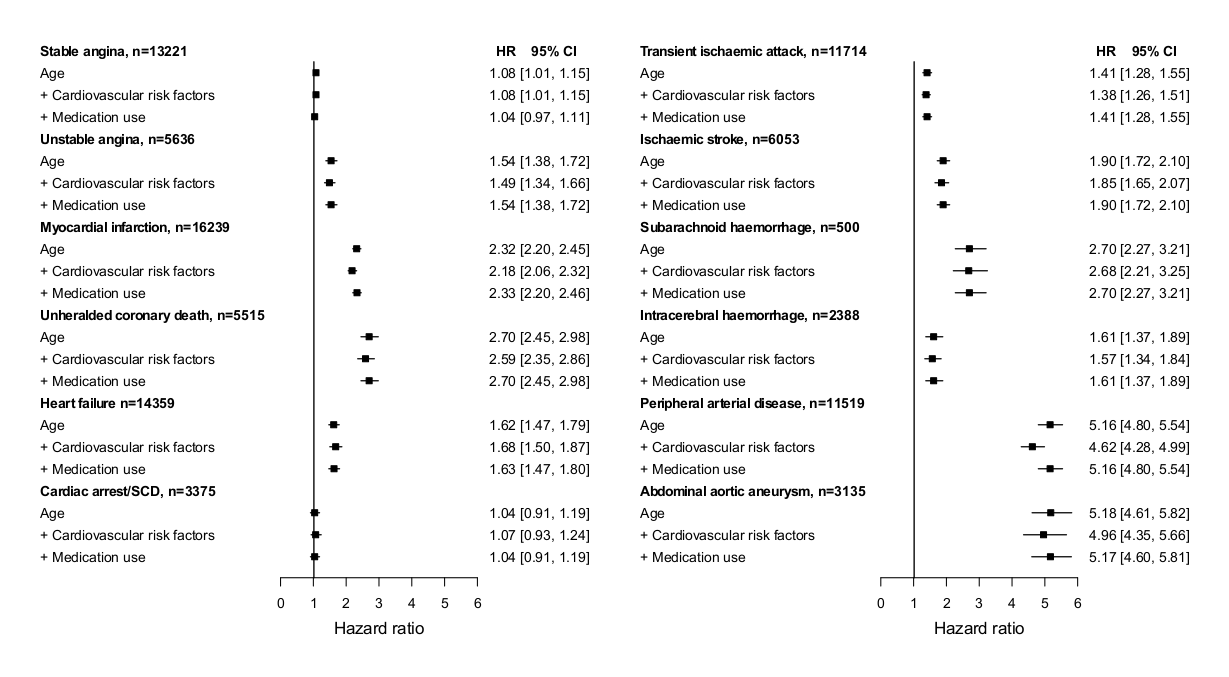


Note: Cardiac arrest/SCD, cardiac arrest, ventricular fibrillation and sudden cardiac death; CI, confidence interval; Cardiovascular risk factors were: body mass index, diabetes, systolic blood pressure, total and high-density lipoprotein cholesterol, and socioeconomic status using the index of multiple deprivation; Medication use included: statins, blood pressure lowering medication, oestrogen oral contraceptives and hormone replacement therapy; HR, age-adjusted hazard ratio from models with baseline hazard function stratified by sex and practice.

2.2.2 Age-adjusted hazard ratios for the association between current vs. never smoking and twelve cardiovascular diseases from observed (complete case analysis) or imputed analyses


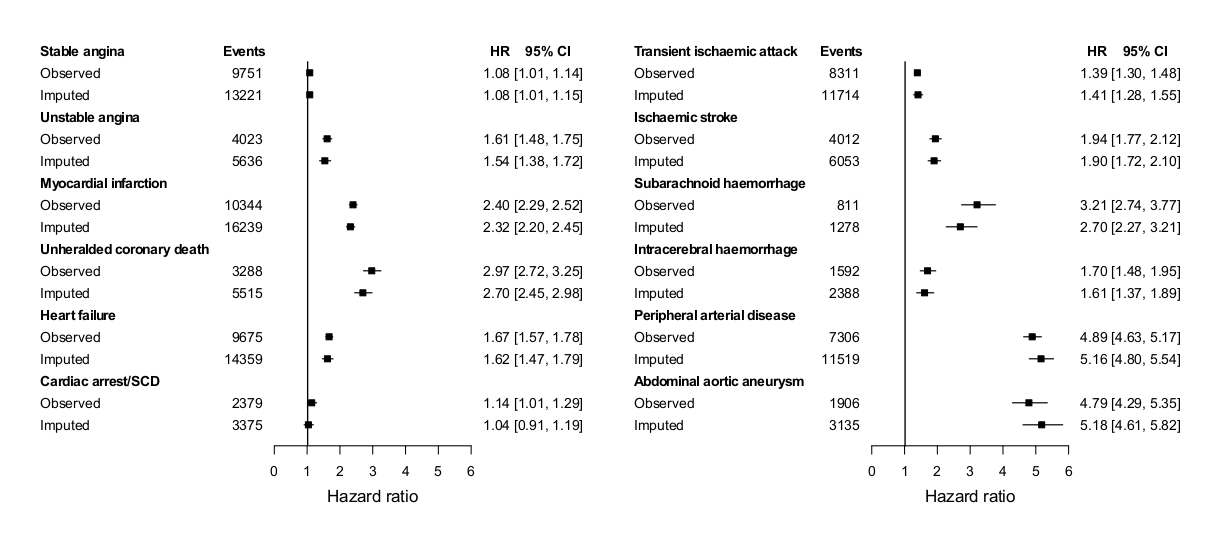


Note: Cardiac arrest/SCD, cardiac arrest, ventricular fibrillation and sudden cardiac death; CI, confidence interval; HR, age-adjusted hazard ratio from models with baseline hazard function stratified by sex and practice. Observed estimates are calculated among patients with recorded data on smoking status (N=1 413 749); imputed estimates are calculated among all patients using observed and imputed smoking status data (as in the primary analysis).

2.2.3 Age-adjusted hazard ratios for the association between current vs. never smoking and twelve cardiovascular diseases by source of endpoint


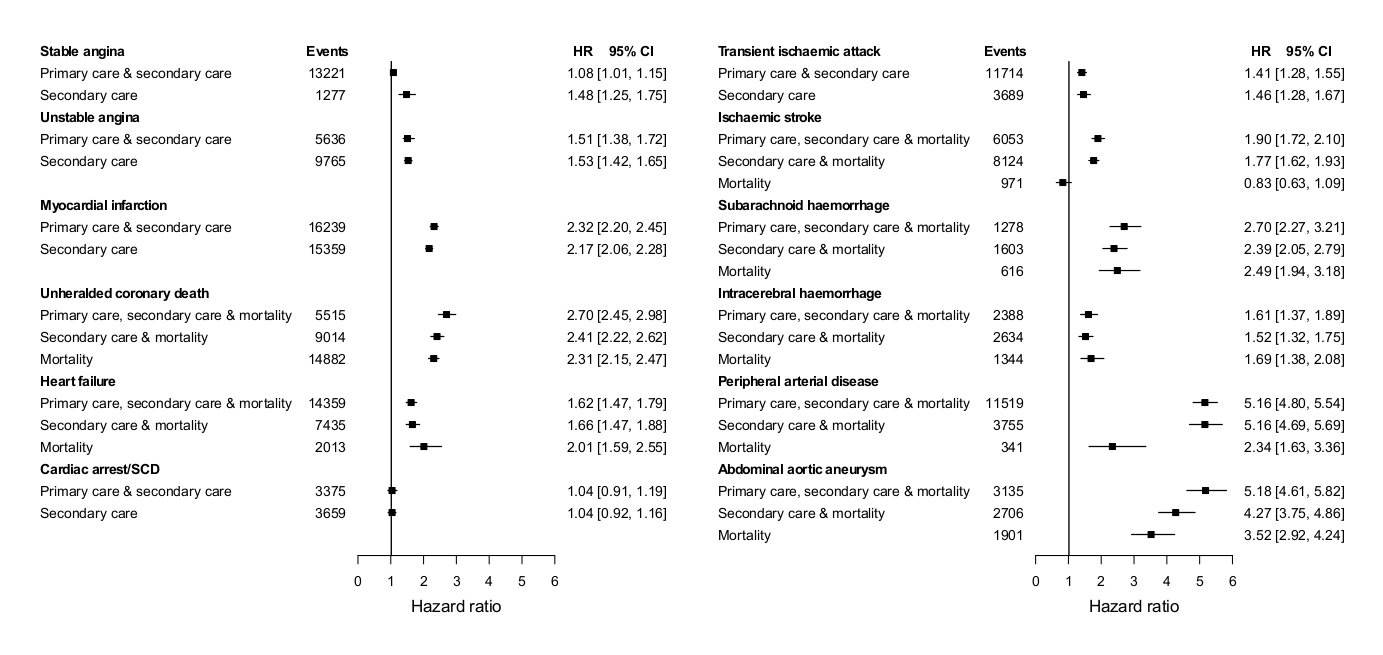


Note: Cardiac arrest/SCD, cardiac arrest, ventricular fibrillation and sudden cardiac death; CI, confidence interval; CVD, cardiovascular diseases; HR, age-adjusted hazard ratio from models with baseline hazard function stratified by sex and practice. Patients with myocardial infarction who died on the date of diagnosis were classified as having an initial presentation with unheralded coronary death.

2.2.4 Age-adjusted hazard ratios for the association between current vs. never smoking and twelve cardiovascular diseases with endpoints defined as initial presentation or first event


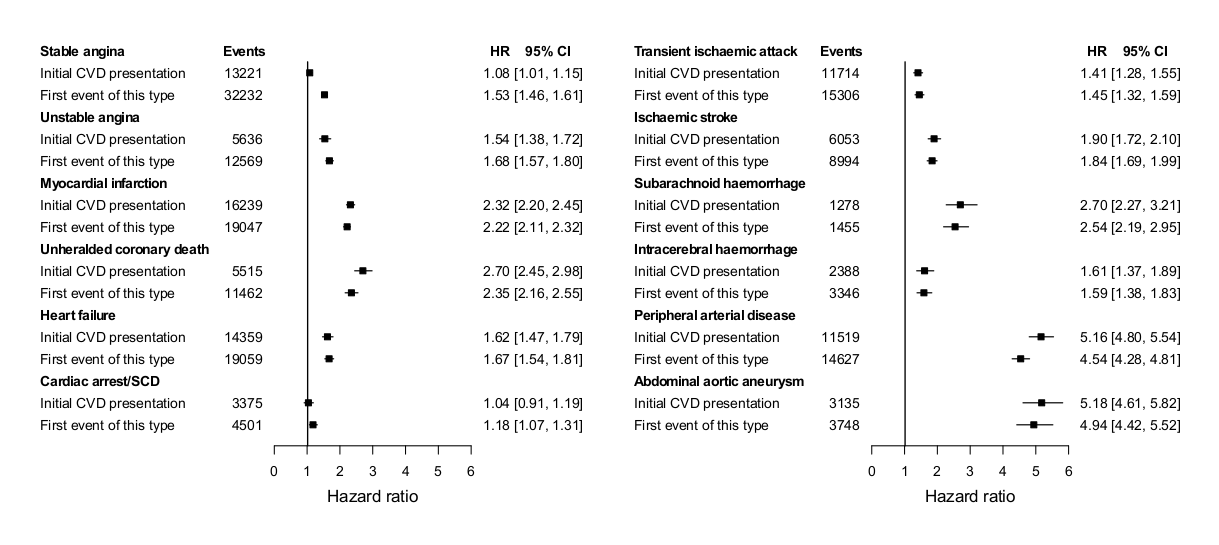


Note: Cardiac arrest/SCD, cardiac arrest, ventricular fibrillation and sudden cardiac death; CI, confidence interval; CVD, cardiovascular diseases; HR, age-adjusted hazard ratio from models stratified by sex and practice.

**2.3 Population attributable fractions with 95% confidence intervals for current and former smoking stratified by sex, based on estimates from models adjusted for age & with baseline hazard function stratified by sex and practice**

|  | **Overall** | | | **Men** | | | **Women** | | |
| --- | --- | --- | --- | --- | --- | --- | --- | --- | --- |
|  | **Current smokers** | **Ex-smokers** | **Total** | **Current smokers** | **Ex-smokers** | **Total** | **Current smokers** | **Ex-smokers** | **Total** |
| **Stable angina** | 2.2 (1.3; 3.1) | 2.7 (2.1; 3.3) | 4.9 | 0.6 (-1.0; 2.1) | 3.6 (2.7; 4.4) | 4.2 | 3.6 (2.5; 4.6) | 1.6 (0.7; 2.6) | 5.2 |
| **Unstable angina** | 6.7 (5.7; 7.6) | 2.2 (1.2; 3.2) | 8.9 | 7.3 (5.8; 8.7) | 3.1 (1.7; 4.5) | 10.4 | 6.1 (4.9; 7.3) | 1.4 (-0.1; 2.9) | 7.5 |
| **Myocardial infarction** | 11.4 (11.1; 11.8) | 3.1 (2.5; 3.6) | 14.5 | 12.2 (11.7; 12.7) | 4.0 (3.3; 4.7) | 16.1 | 11.0 (10.6; 11.5) | 2.0 (1.1; 3.0) | 13.1 |
| **Unheralded coronary death** | 12.0 (11.4; 12.5) | 3.9 (3.0; 4.7) | 15.9 | 14.3 (13.6; 15.0) | 4.2 (2.9; 5.4) | 18.5 | 8.7 (7.5; 9.8) | 4.4 (3.3; 5.5) | 13.1 |
| **Heart failure** | 8.3 (7.7; 8.9) | 3.1 (2.6; 3.7) | 11.4 | 7.9 (6.8; 9.0) | 3.7 (2.8; 4.5) | 11.6 | 8.3 (7.6; 8.9) | 2·6 (1.9; 3.3) | 10.9 |
| **Cardiac arrest/SCD** | 2.0 (0.2; 3.8) | 2.3 (1.1; 3.5) | 4.3 | 1.4 (-1.3; 4.0) | 1.8 (0.1; 3.5) | 3.2 | 2.9 (0.4; 5.3) | 3.1 (1.4; 4.9) | 6.0 |
| **Transient ischaemic attack** | 6.3 (5.5; 7.0) | 0.7 (-0.1; 1.4) | 3.9 | 6.7 (5.5; 7.9) | 0.6 (-0.6; 1.8) | 7.3 | 5.7 (4.8; 6.6) | 0.6 (-0.4; 1.6) | 6.4 |
| **Ischaemic stroke** | 10.0 (9.3; 10.7) | 2.2 (1.2; 3.1) | 12.2 | 12.0 (10.9; 13.0) | 2.3 (0.8; 3.7) | 14.2 | 8.1 (7.1; 9.1) | 2.4 (1.2; 3.6) | 10.5 |
| **Subarachnoid haemorrhage** | 12.5 (11.5; 13.4) | 2.5 (0.1; 4.8) | 15.0 | 14.2 (12.5; 15.9) | 0.1 (-5.0; 4.8) | 14.3 | 10.8 (9.7; 12.0) | 3.5 (0.1; 5.8) | 14.3 |
| **Intracerebral haemorrhage** | 8.5 (7.2; 9.9) | 2.4 (0.9; 3.9) | 10.9 | 7.6 (5.2; 10.0) | 1.0 (-1.6; 3.5) | 8.6 | 8.8 (7.4; 10.2) | 3.4 (1.7; 5.1) | 12·2 |
| **Peripheral arterial disease** | 16.3 (16.1; 16.5) | 6.0 (5.4; 6.5) | 22.3 | 18.9 (18.7; 19.1) | 8.1 (7.4; 8.8) | 27.0 | 13.7 (13.5; 14.0) | 4.1 (3.2; 4.9) | 17.8 |
| **Abdominal aortic aneurysm** | 16.3 (16.0; 16.6) | 5.3 (4.2; 6.3) | 21.6 | 18.5 (18.1; 18.9) | 7.0 (5.8; 8.3) | 25.6 | 13.9 (13.2; 14.5) | 1.9 (-0.6; 4.3) | 15.7 |
| **CVD** | 10.6 (10.5; 10.8) | 2.6 (2.4; 2.9) | 13.2 | 11.9 (11.7; 12.17) | 3.5 (3.2; 3.8) | 15.4 | 9.3 (9.1; 9.5) | 1.9 (1.6; 2.3) | 11.2 |

Note: Cardiac arrest/SCD, cardiac arrest, ventricular fibrillation and sudden cardiac death.

**2.4 Interactions with sex, age, diabetes and hypertension**

2.4.1 Hazard ratios for the association between current vs. never smokers and twelve cardiovascular diseases by baseline age group in men and women


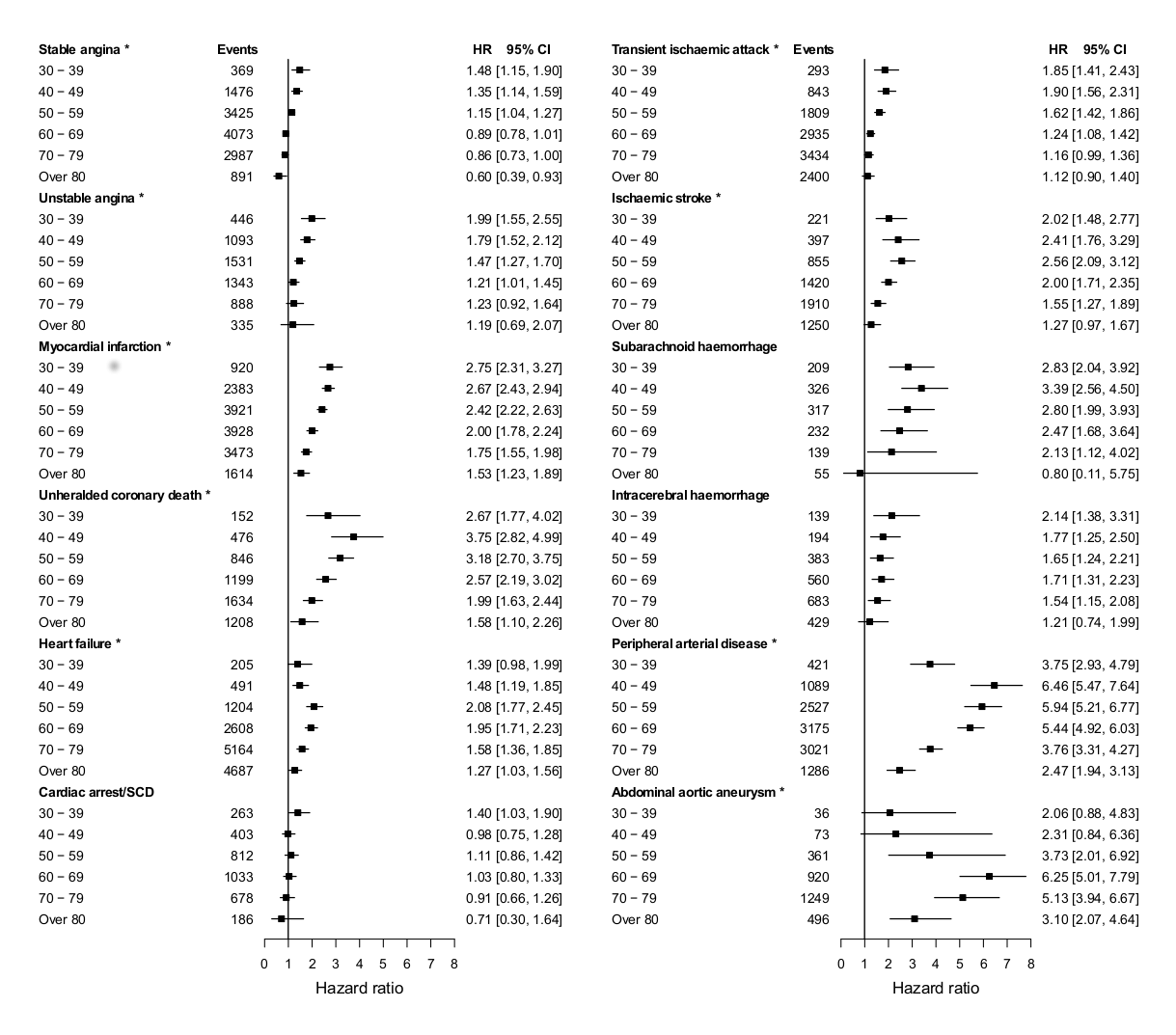


Note: Cardiac arrest/SCD, cardiac arrest, ventricular fibrillation and sudden cardiac death; CI, confidence interval; HR, hazard ratio from models with baseline hazard function stratified by sex and practice. * P-value for interaction <0.05 (*P*=0.0005 for unstable angina and *P*<0.0001 for the other 8 cardiovascular diseases).

2.4.2 Hazard ratios for the association between current smoking and twelve cardiovascular diseases by baseline age group in men


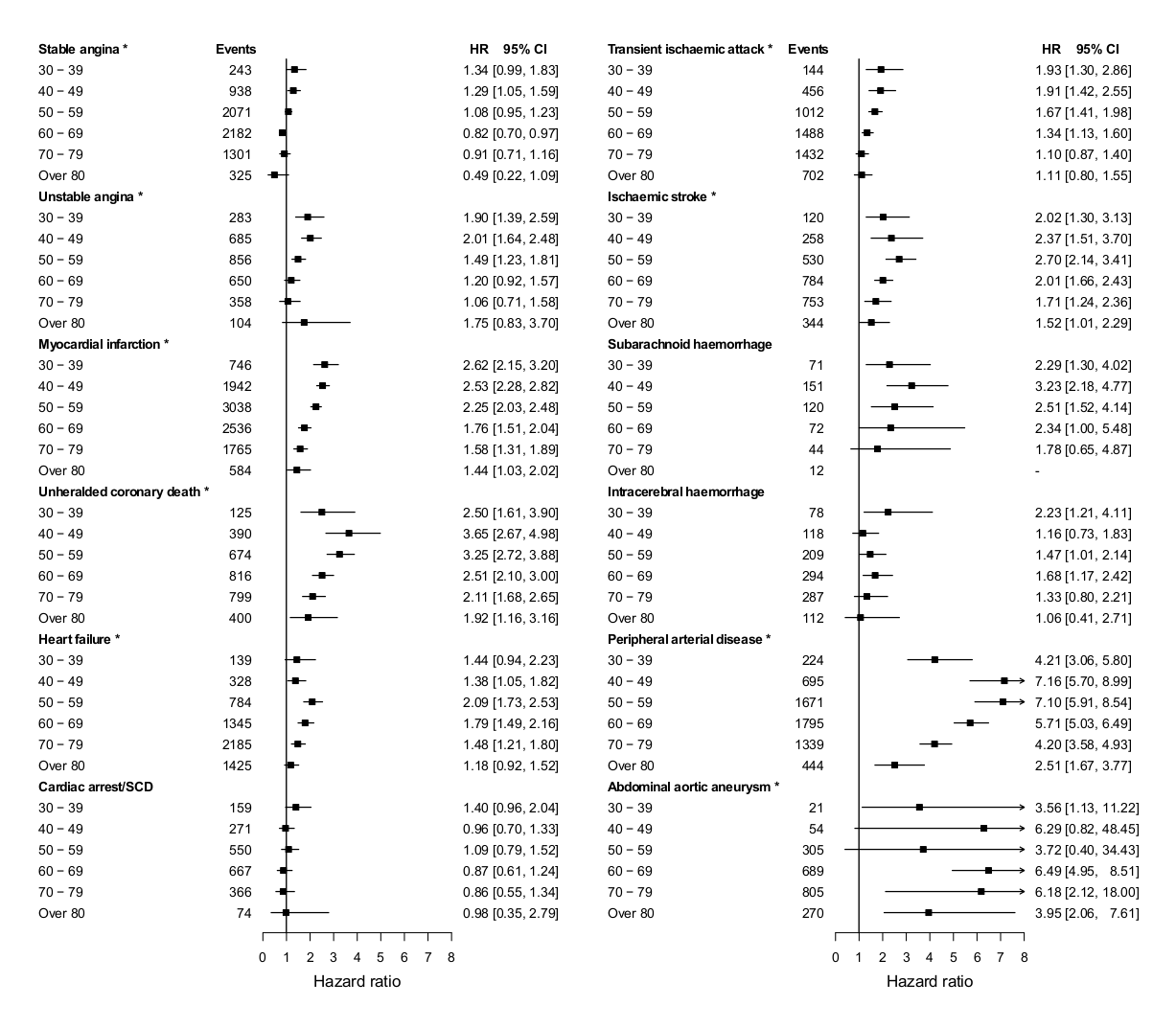


Note: Cardiac arrest/SCD, cardiac arrest, ventricular fibrillation and sudden cardiac death; CI, confidence interval; HR, hazard ratio from models with baseline hazard function stratified by sex and practice. *P-value for interaction <0.05 (*P*=0.02 for abdominal aortic aneurysm, *P*=0.01 for ischaemic stroke, *P*=0.003 for unstable angina, *P*=0.0005 for unheralded coronary death, and *P*<0.0001 for the other 5 cardiovascular diseases).

2.4.3 Hazard ratios for the association between current smoking and twelve cardiovascular diseases by baseline age group in women


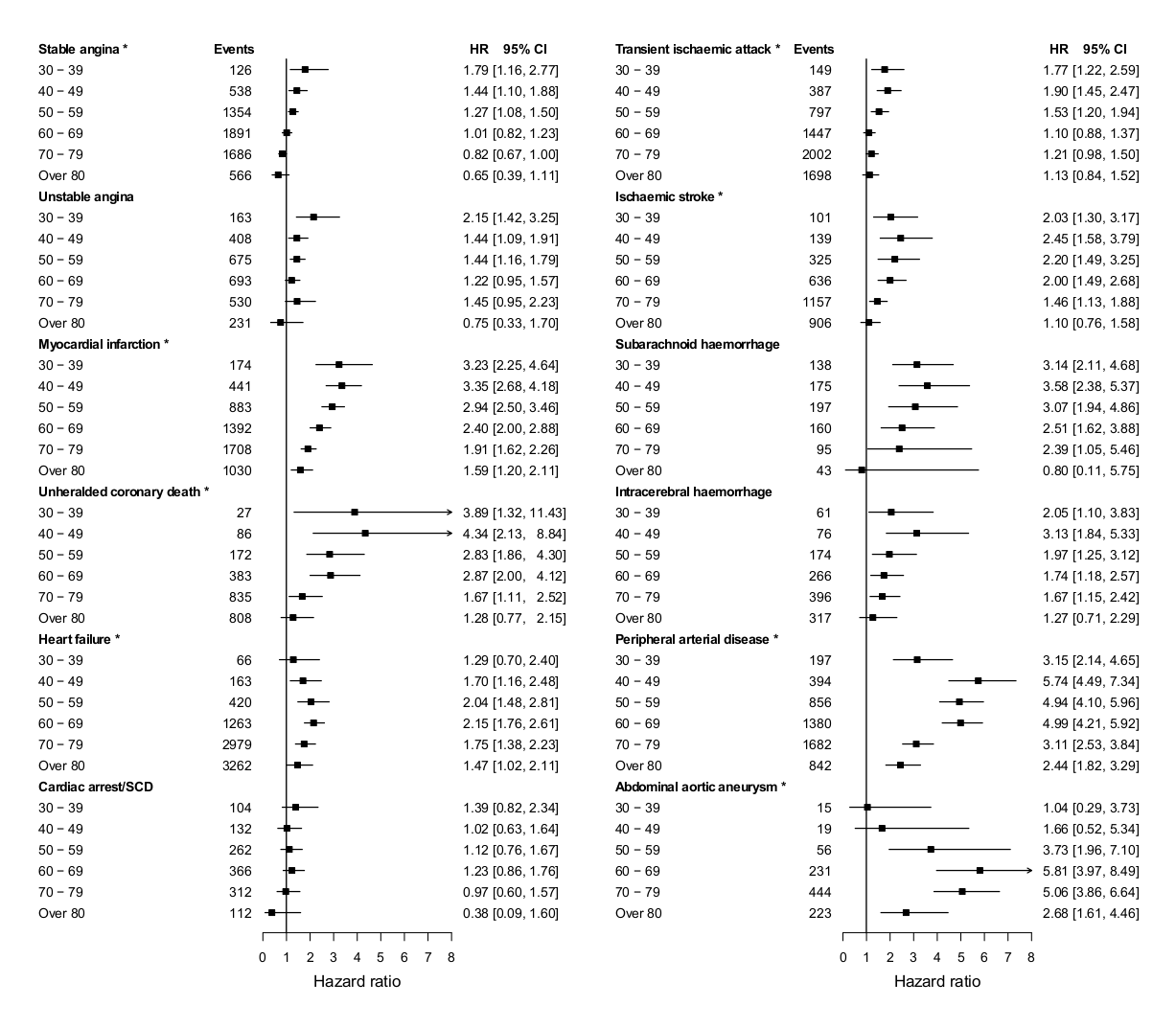


Note: Cardiac arrest/SCD, cardiac arrest, ventricular fibrillation and sudden cardiac death; CI, confidence interval; HR, hazard ratio from models with baseline hazard function stratified by sex and practice. *P-value for interaction <0.05 (*P*<0.009 for ischaemic stroke, *P*=0.0007 for abdominal aortic aneurysm, and *P*<0.0001 for the other 6 cardiovascular diseases).

2.4.4 Age-adjusted hazard ratios for the association between current vs. never smoking and twelve cardiovascular diseases in patients with or without diabetes


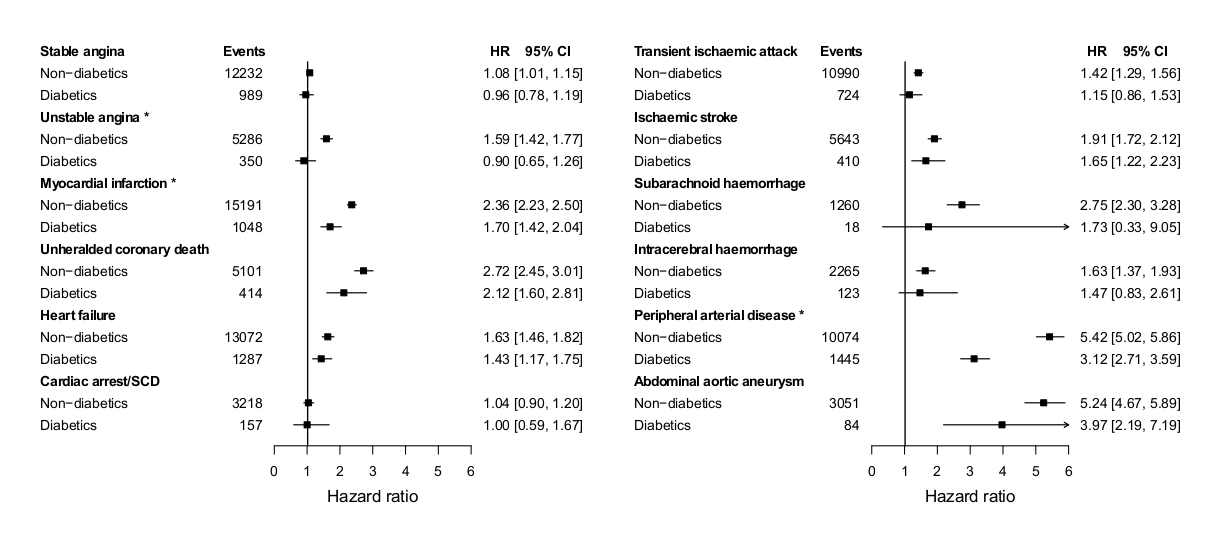


Note: Cardiac arrest/SCD, cardiac arrest, ventricular fibrillation and sudden cardiac death; CI, confidence interval; HR, age-adjusted hazard ratio from models with baseline hazard function stratified by sex and practice. *P-value for interaction <0.05 (*P*=0.04 for unstable angina, *P*<0.0001 for peripheral arterial disease, and *P*=0.001 for myocardial infarction).

2.4.5 Age-adjusted hazard ratios for the association between current vs. never smoking and twelve cardiovascular diseases in patients with or without hypertension


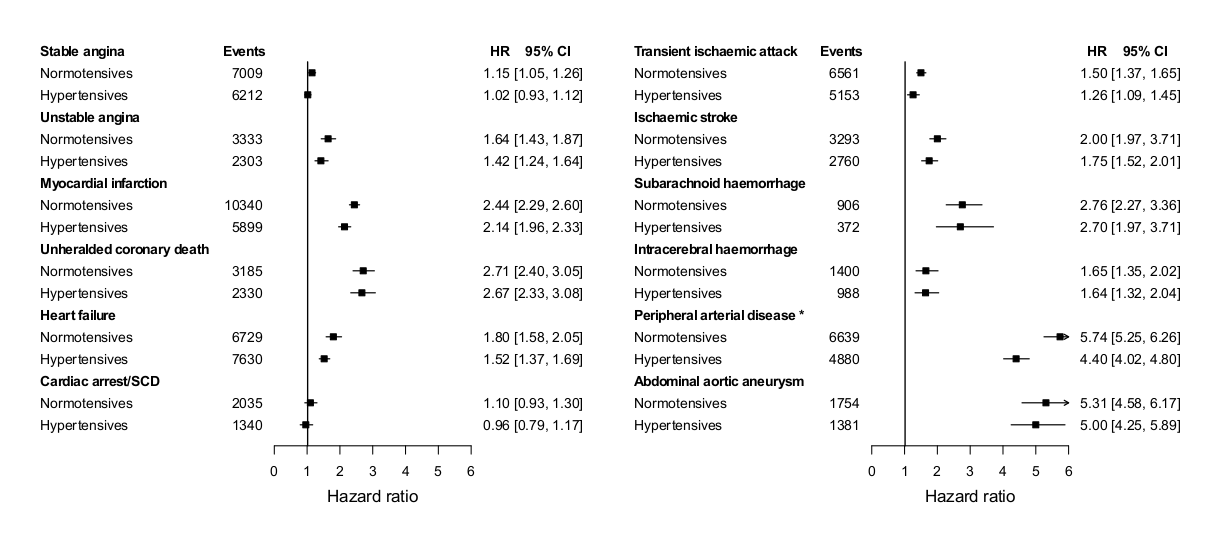


Note: Cardiac arrest/SCD, cardiac arrest, ventricular fibrillation and sudden cardiac death; CI, confidence interval; HR, age-adjusted hazard ratio from models with baseline hazard function stratified by sex and practice. *P-value for interaction <0.05 (*P*=0.004 for peripheral arterial disease).

**2.5 Age-adjusted hazard ratios for the association between duration of smoking cessation and twelve cardiovascular diseases (ref. never smokers)**


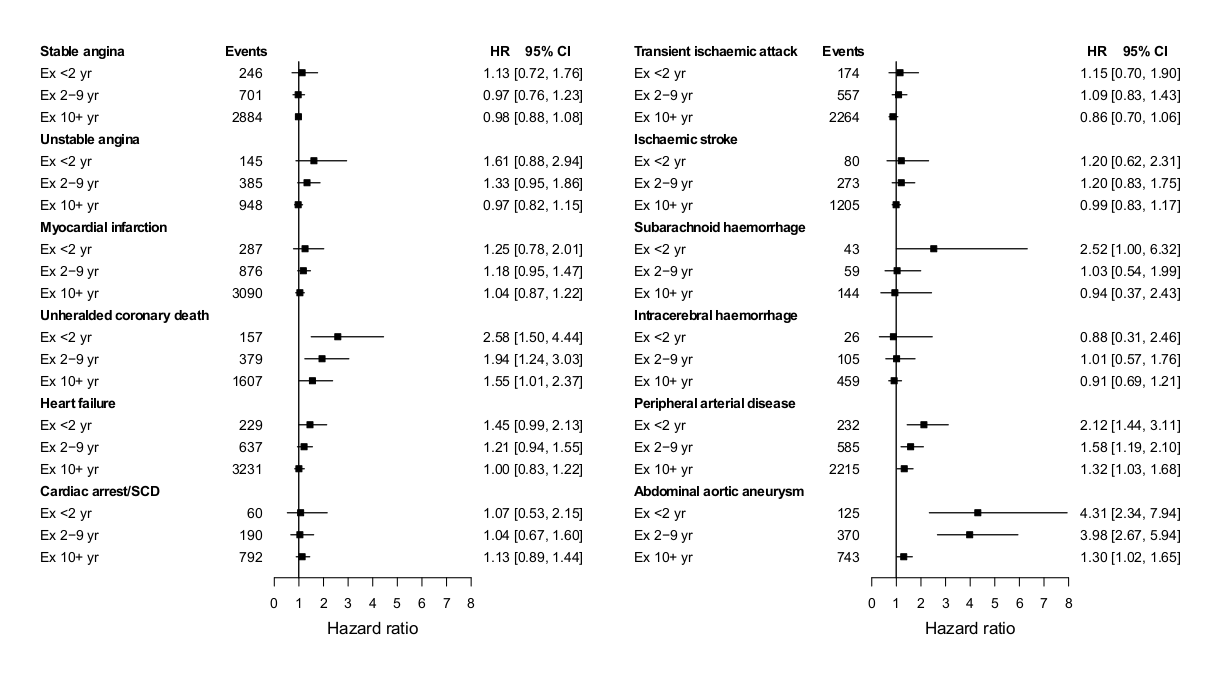


Note: Cardiac arrest/SCD, cardiac arrest, ventricular fibrillation and sudden cardiac death; CI, confidence interval; HR, age-adjusted hazard ratio from models with baseline hazard function stratified by sex and practice.

**2.6 Public health interventions**

2.6.1 Age-adjusted hazard ratios for the association between current vs. never smoking and twelve cardiovascular diseases in the period before and after the introduction of financial reward for recording of smoking data (April 2004)


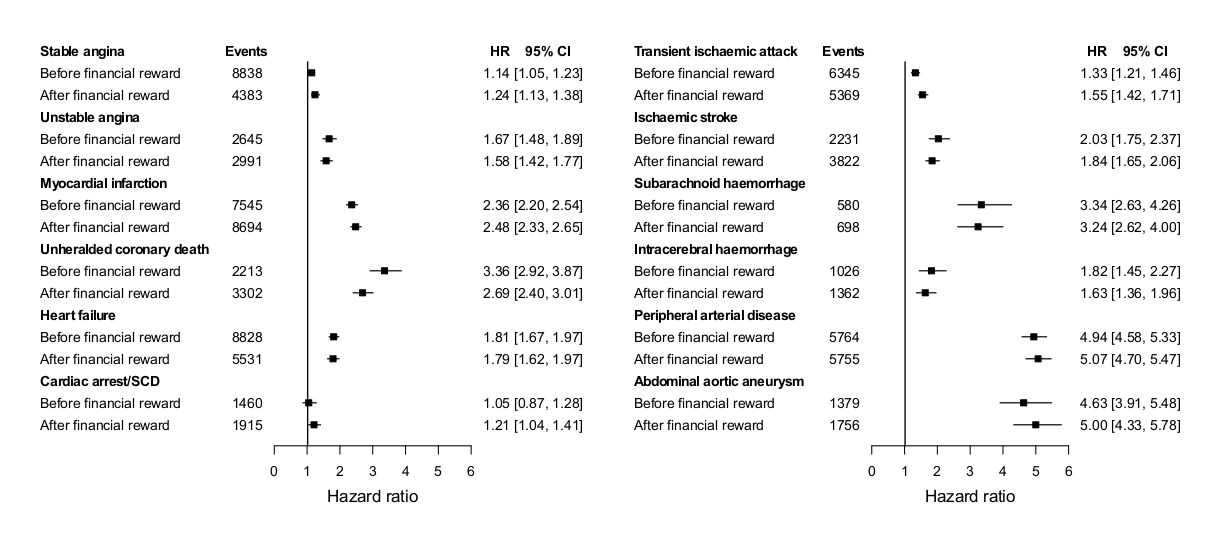


Note: Cardiac arrest/SCD, cardiac arrest, ventricular fibrillation and sudden cardiac death; CI, confidence interval; HR, age-adjusted hazard ratio from models with baseline hazard function stratified by sex and practice.

2.6.2 Age-adjusted hazard ratios for the association between current vs. never smoking and twelve cardiovascular diseases in the period before and after implementation of the smoke-free legislation in England (January 2007)


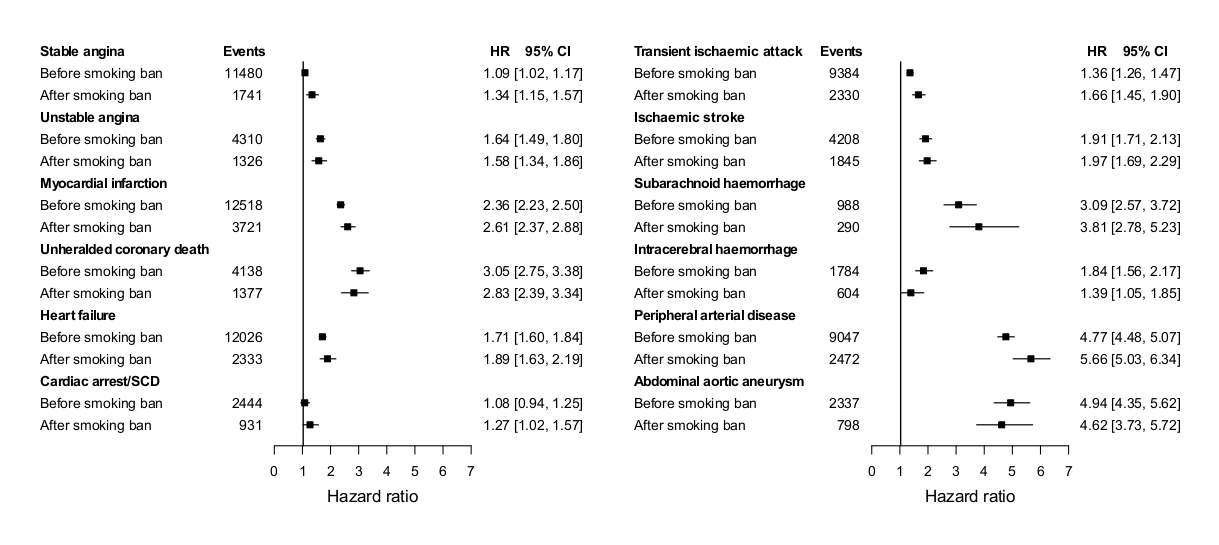


Note: Cardiac arrest/SCD, cardiac arrest, ventricular fibrillation and sudden cardiac death; CI, confidence interval; HR, age-adjusted hazard ratio from models with baseline hazard function stratified by sex and practice.
